# Sustained efficacy of the RTS,S/AS01_E_ malaria vaccine over 50 months of follow-up when used in full-dose or fractional-dose regimens in young children in Ghana and Kenya: final results from an open-label, phase 2b, randomised controlled trial

**DOI:** 10.1016/S2214-109X(25)00272-4

**Published:** 2025-10

**Authors:** Lawrence Osei-Tutu, Simon K Kariuki, Cynthia K Lee, Robin Fabre, Dennis K Bii, Samuel Adjei, Martina Oneko, Maame Anima Attobrah Sarfo, Christian F Ockenhouse, Lode Schuerman, Patrick Boakye Yiadom Buabeng, Ashura Bakari, Cecilia Atieno, Maame Fremah Kotoh-Mortty, Kephas Otieno, Yaw Ntiamoah, Tony Sang, Anne Bollaerts, Nelli Westercamp, Daniel Ansong, Tsiri Agbenyega, Aaron M Samuels, Opokua Ofori-Anyinam

**Affiliations:** Kwame Nkrumah University of Science and Technology/Agogo Presbyterian Hospital, Agogo, Asante Akyem, Ghana (L Osei-Tutu MD, S Adjei MD, M A Attobrah Sarfo MD, P B Y Buabeng MBA, A Bakari MD, M F Kotoh-Mortty MD, Y Ntiamoah MPhil, Prof D Ansong MD, Prof T Agbenyega MD); **Centre for Global Health Research, Kenya Medical Research Institute, Kisumu, Kenya** (S K Kariuki PhD, D K Bii MD, M Oneko MD, C Atieno BScN, K Otieno MSc, T Sang BPharm); **PATH’s Center for Vaccine Innovation and Access, Washington, DC, USA** (C K Lee PhD, C F Ockenhouse MD); **GSK, Wavre, Belgium** (R Fabre MSc‡, L Schuerman MD‡, A Bollaerts MSc, O Ofori-Anyinam PhD); **Malaria Branch, Division of Parasitic Diseases and Malaria, National Center for Emerging Zoonotic Infectious Diseases, Centers for Disease Control and Prevention, Atlanta, GA, USA** (N Westercamp PhD, A M Samuels MD); **Malaria Branch, Division of Parasitic Diseases and Malaria, National Center for Emerging Zoonotic Infectious Diseases, US Centers for Disease Control and Prevention, Kisumu, Kenya** (A M Samuels MD)

## Abstract

**Background:**

We conducted a phase 2b trial evaluating fractional-dose and full-dose regimens of the RTS,S/AS01_E_ vaccine (RTS,S). All regimens provided substantial protection against clinical malaria in natural exposure settings, over 21 and 32 months of follow-up. Here, we present end-of-study results, after 50 months of follow-up.

**Methods:**

This open-label, randomised controlled trial was conducted at two research centres in Agogo (Ghana) and Siaya County (Kenya) between Sept 28, 2017, and Nov 14, 2022. Children aged 5–17 months were randomly assigned (1:1:1:1:1) to one of five groups to receive rabies vaccine (the control group) at months 0, 1, and 2; or full doses of RTS,S at months 0, 1, and 2, followed by either full doses (R) at month 20 (group R012-20) or months 14, 26, and 38 (R012-14-26-38); or full doses at months 0 and 1, followed by fractional doses (Fx; one-fifth of full dose) at months 2, 14, 26, and 38 (Fx012-14-26-38) or months 7, 20, and 32 (Fx017-20-32). We present results of secondary objectives, evaluating vaccine efficacy, impact, immunogenicity, and harms up to month 50. Endpoints were the occurrence of clinical malaria meeting the primary and secondary case definitions and antibody responses at predefined timepoints, and the occurrence of solicited adverse events within 7 days from vaccination and serious adverse events and adverse events of special interest up to study end. This trial is registered at ClinicalTrials.gov (NCT03276962) and is complete.

**Findings:**

Between Sept 28, 2017, and Sept 25, 2018, 2157 children were enrolled, of whom 1609 were randomly assigned (322 to each RTS,S group and 321 to the control group). Of these 1609 children, 1500 received at least one study vaccine dose (exposed set), and 1333 were included in the per-protocol set for efficacy. Among children in the exposed set, to month 50, vaccine efficacy against all episodes of clinical malaria was 36% (95% CI 19–50), 51% (37–61), 43% (28–55), and 41% (26–53) in groups R012-20, R012-14-26-38, Fx012-14-26-38, and Fx017-20-32, respectively (p<0·001 for all). The numbers of cases averted per 1000 RTS,S full-dose equivalents were 353 (R012-20 group), 544 (R012-14-26-38 group), 1151 (Fx012-14-26-38 group), and 1134 (Fx017-20-32 group). Vaccine efficacy and impact and immune responses were maintained over 50 months of follow-up in groups who received additional vaccine doses after the fourth dose. The vaccine was well tolerated; only five serious adverse events were considered to be related to vaccination. There were no deaths considered to be related to vaccination.

**Interpretation:**

All RTS,S regimens provided substantial protection against clinical malaria, with additional yearly doses maintaining vaccine efficacy and impact up to 50 months. Using fractional-dose regimens could increase the availability of RTS,S and reduce vaccination cost.

**Funding:**

GSK; PATH (through the Bill & Melinda Gates Foundation and the German Federal Ministry of Education and Research).

## Introduction

In the last 5 years, we have witnessed the achievement of several important milestones in the fight against malaria. The pilot introduction of the RTS,S/AS01_E_ vaccine (RTS,S) in the national immunisation programmes of Ghana, Kenya, and Malawi between 2019 and 2023 was evaluated through the Malaria Vaccine Programme Evaluation (MVPE).^1^ Following 24 months of piloting, citing the MVPE results^2^ as key evidence, WHO recommended implementation of RTS,S in areas of moderate-to-high malaria transmission.^3^ MVPE results after 4 years showed a 13% reduction in mortality from all causes and a 32% reduction in severe malaria hospitalisations among children younger than 5 years.^1,2^ Two malaria vaccines—RTS,S since July, 2022, and R21/Matrix-M since December, 2023—are now prequalified by WHO.^1^ Since October, 2023, WHO has recommended the programmatic use of either vaccine for the prevention of *Plasmodium falciparum* malaria in children from all malaria-endemic regions, prioritising areas of moderate and high transmission.^3,4^

RTS,S is administered on a four-dose schedule from 5 months of age, with at least 4 weeks between the first three doses, and a fourth dose administered 12–18 months after the third dose. However, flexibility in the timing of administrations is allowed for optimised delivery by allowing alignment with routine immunisation schedules and local programme logistics. In areas with highly seasonal malaria or those with perennial transmission and seasonal peaks, seasonal five-dose schedules are also recommended, with additional doses to be provided annually, before peak transmission season.^5^

In a phase 2b trial, we evaluated the efficacy of different fractional-dose (Fx) and full-dose (R) RTS,S regimens in children in natural exposure settings. The primary objective was to evaluate whether vaccine efficacy over 12 months post-dose 3 for a fractional regimen with a third fractional dose (Fx012) was superior than a full-dose regimen (R012). Although we did not find superiority,^6^ we showed that all RTS,S regimens provided substantial protection against clinical malaria, which tended to be similar up to 32 months after the first vaccination, thus demonstrating potential flexibility in dosing regimen and schedule.^7^ Cases of clinical malaria averted per 1000 children vaccinated were also similar between fractional-dose and full-dose regimens over 21 months^6^ and 32 months^7^ of follow-up after the first vaccination. In addition, when accounting for full-dose equivalence, fractional-dose regimens led to more cases averted than full-dose regimens.^7^

Many countries are now integrating malaria vaccines into their routine childhood vaccination programmes.^8^ Should fractional-dose RTS,S regimens prove to be as efficacious as standard full-dose regimens, their use could increase access to vaccination and might improve cost-effectiveness of mass immunisation programmes through reductions in production costs, as previously recommended by WHO for other vaccines (eg, against poliomyelitis, yellow fever, or rabies^9-11^). Fractional RTS,S doses could also be used for multiple vaccinations administered yearly, to extend the period of protection against malaria in children by ensuring optimal vaccine efficacy over several years. Here, we report the final results of the phase 2b trial, after 50 months of follow-up post-first vaccination with fractional-dose or full-dose RTS,S regimens.

## Methods

### Study design and participants

The study design has previously been described in detail^6^ and is summarised in appendix 1 (p 1). Briefly, we conducted an open-label, phase 2b, randomised controlled trial at the Malaria Research Center, Agogo, Ashanti Region (Ghana), and the Centre for Global Health Research of the Kenya Medical Research Institute (KEMRI) and the US Centers for Disease Control and Prevention site in Siaya County (Kenya). The study was conducted between Sept 28, 2017, and Nov 14, 2022. After obtaining informed consent, we enrolled children aged 5–17 months without serious acute or chronic illness and who previously received three doses of diphtheria, pertussis, tetanus, and hepatitis B vaccine and at least three doses of oral polio vaccine.

The trial protocol and informed consent form were approved by local and national regulatory authorities and institutional review boards or independent ethics committees (appendix 1 p 2). An independent data monitoring committee oversaw the study. The trial was conducted in accordance with the principles of the Declaration of Helsinki. The trial was registered with ClinicalTrials.gov (NCT03276962) and is complete.

### Randomisation and masking

Children were randomly assigned (1:1:1:1:1) to one of five groups receiving either one of four RTS,S regimens or a control vaccine against rabies. The rabies vaccine^12^ was administered at months 0, 1, and 2. All RTS,S groups received two full RTS,S doses at months 0 and 1, followed by either full doses at months 2 and 20 (group R012-20 [standard regimen]), full doses at months 2, 14, 26, and 38 (group R012-14-26-38), fractional doses at months 2, 14, 26, and 38 (group Fx012-14-26-38), or fractional doses at months 7, 20, and 32 (group Fx017-20-32). Randomisation was performed using a web-based randomisation system with a minimisation procedure accounting for trial centre (appendix 1 p 2). The trial was open-label, with no masking of participants or outcome assessors.

### Procedures

The composition of RTS,S was previously described in detail.^13^ Fractional doses were administered as one-fifth (0·1 mL) of the full RTS,S dose (0·5 mL) after reconstitution. Vaccines were administered by intramuscular injection in the left deltoid.

Study procedures have been previously described.^6^ Briefly, malaria cases were captured via passive detection; parents or guardians were asked to bring the child to the study-designated clinic in case of illness. The primary case definition for clinical malaria was *P falciparum* asexual parasitaemia above 5000 parasites per μL of blood and fever (axillary temperature ≥37·5°C). The secondary case definition was any *P falciparum* asexual parasitaemia (>0 parasites per μL) and fever or history of fever within 24 h of presentation.

The prevalence of *P falciparum* infections was assessed at each cross-sectional visit (each calendar month up to month 20 and every 3 months thereafter). Blood samples for assessment of immunogenicity^6^ were collected as indicated in appendix 1 (p 1).

Solicited local and general adverse events were collected within 4 days post-vaccination (by trained site personnel) and unsolicited adverse events within 30 days post-vaccination. Cases of severe malaria and cerebral malaria, serious adverse events, adverse events of specific interest (meningitis and potential immune-mediated diseases), and adverse events leading to study withdrawal were collected throughout the study.

### Outcomes

We have previously reported findings for the primary endpoint (occurrence of clinical malaria meeting the primary case definition at 12 months after the third vaccination) and interim results for secondary endpoints up to 33 months of follow-up.^6,7^ Here, we present other secondary endpoints, including the occurrence of clinical malaria meeting primary and secondary case definitions over different follow-up periods (ie, from study start to month 50 and over 12 months after each additional yearly vaccine dose) and the prevalence and incidence of *P falciparum* infections. We also present antibody responses to the *P falciparum* circumsporozoite protein and hepatitis B surface antigen and harms data up to month 50.

### Statistical analysis

Sample size considerations have previously been presented.^6^ Vaccine efficacy estimates against all episodes of clinical malaria were calculated with 95% CIs as 100 × (1 – incidence rate ratio), overall (adjusted for country as a fixed effect) and by country, and analysed by negative binomial regression allowing for interdependence between episodes within the same child.^14^ Vaccine efficacy was calculated for RTS,S groups compared with the control group; incremental vaccine efficacy estimates were calculated by comparing the RTS,S groups with each other.

We calculated the estimated number of cases as the area under the 3-month incidence curve of clinical malaria for each group. We then defined vaccine impact as the estimated number of cases of clinical malaria (according to the secondary case defintion) averted over the relevant period per 1000 children vaccinated (sum of the differences in incidence between RTS,S and control groups per 3-month periods multiplied by 1000/4). To estimate the cumulative cases averted per 1000 full-dose equivalents of vaccine doses administered (eg, a fractional dose corresponded to 0·2 full-dose equivalent RTS,S doses), the cumulative number of cases averted per 1000 children vaccinated was divided by the average number of doses received until each considered timepoint by participants in each group (post-hoc analysis). The average number of doses was calculated in each group as the total number of full-dose equivalents received until that timepoint by all participants who contributed to malaria surveillance since the month 0–3 period, divided by the corresponding number of participants. We also estimated the effect of the additional yearly vaccine doses, by subtracting the cumulative number of cases of clinical malaria averted (secondary case definition) occurring up to month 21 from the total number of cumulative cases (post-hoc analysis).

Vaccine efficacy and impact analyses were carried out in the per-protocol set for efficacy (including children who received all three first vaccinations as per protocol and contributed to efficacy surveillance starting 14 days post-dose 3) or the exposed set (children who received at least one vaccination). In post-hoc analyses in the exposed set, we also estimated vaccine efficacy over 50 months of follow-up in three age categories at first vaccination (5–8, 9–12, and ≥13 months) and in children with positive or negative baseline parasitaemia, based on microscopy.

Immunogenicity and reactogenicity analyses were conducted in a subset of 250 children (ie, the first 50 children randomly assigned into each group at each site), while all other analyses of harms were conducted in the exposed set.

All analyses were performed with SAS 9.4. Participants withdrawn or lost to follow-up were not replaced; missing data were not imputed.

### Role of the funding source

GSK was involved in study design and oversight; coordinated data collection, analysis, and interpretation; and was involved in the writing of the report. PATH’s Center for Vaccine Innovation and Access contributed to study design and data interpretation but was not involved in data collection.

## Results

**Figure 1:**
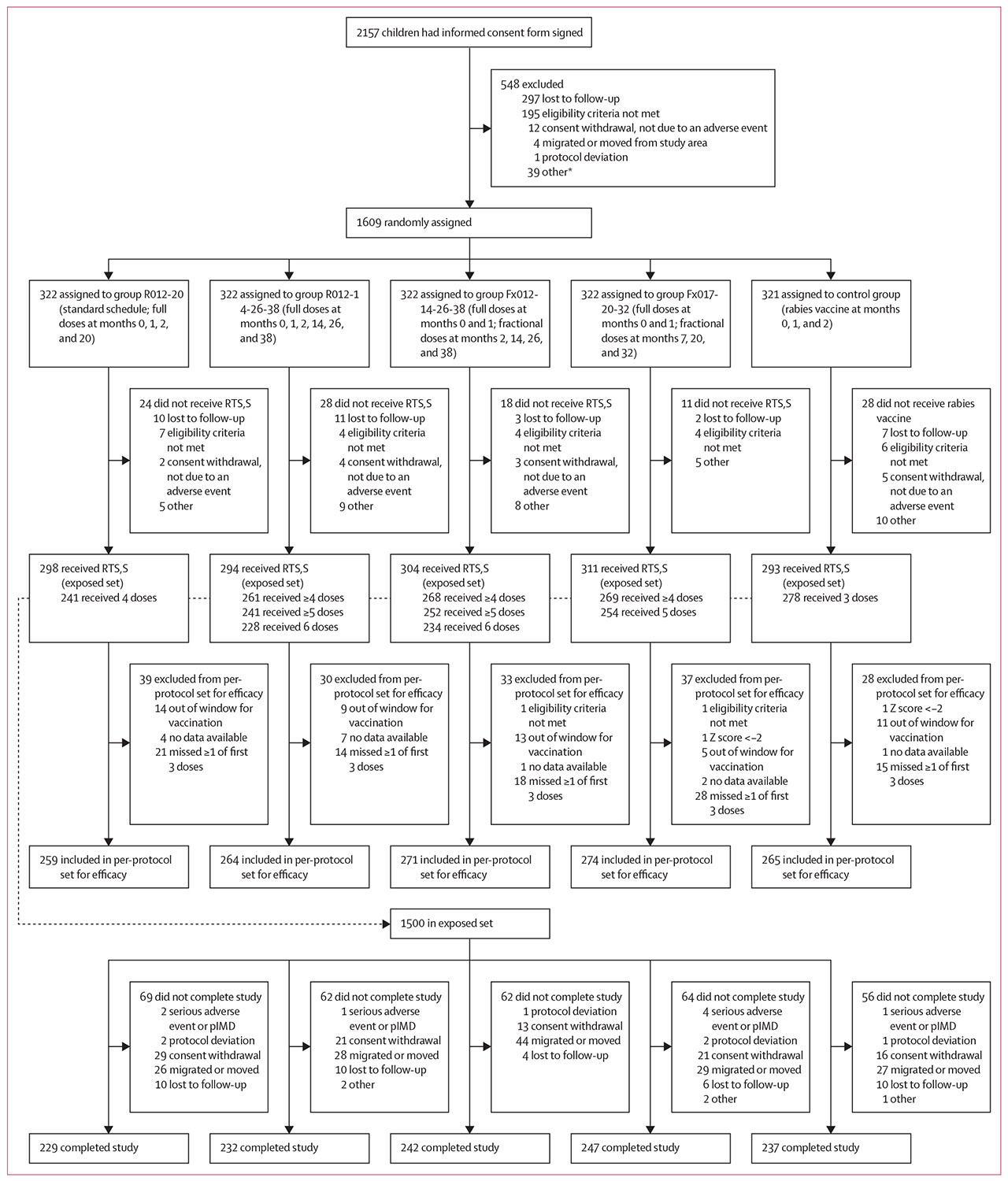
Trial profile pIMD=potential immune-mediated disease. *Other reasons for exclusion included: not attending first visit as scheduled (within 28 days from screening), incomplete screening procedures, one parent declining participation, Z score less than −2, low haemoglobin concentration, moderate malnutrition, and recruitment target reached.

Between Sept 28, 2017, and Sept 25, 2018, 2157 children were enrolled following consent from parent(s) or legally accepted representative(s). In total, 1609 children were randomly assigned to a treatment group, 1500 received at least one vaccine dose and were included in the exposed set, 1333 were included in the per-protocol set for efficacy, and 1187 completed the study (figure 1). Participant characteristics, previously reported in detail,^6,7^ were similar between groups. Across groups, the mean age at first vaccination was 10·2–10·5 months. A similar proportion of boys and girls were enrolled in all groups except for R012-20, in which more boys (60·1%) than girls (39·9%) were enrolled.

**Figure 2:**
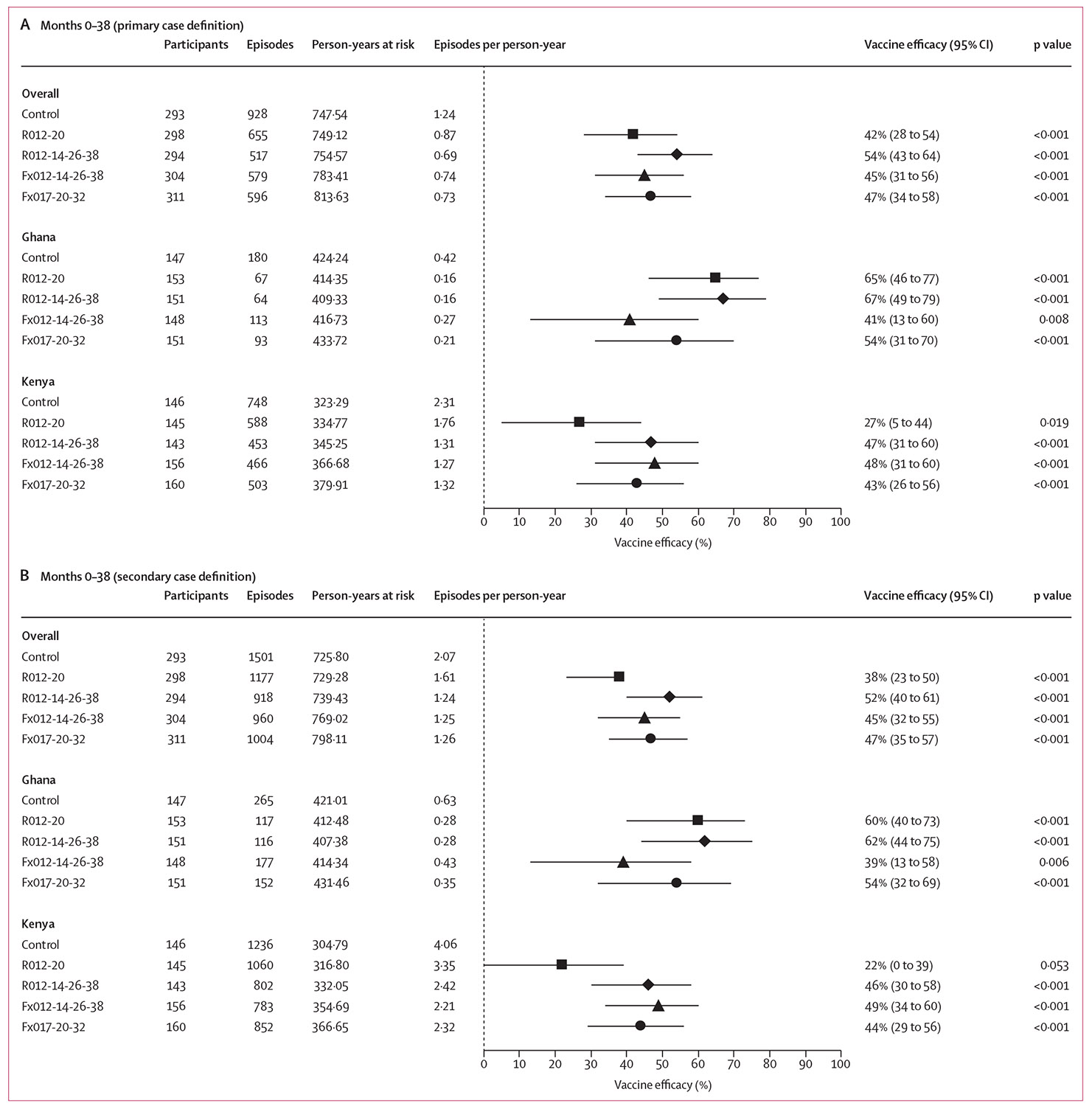

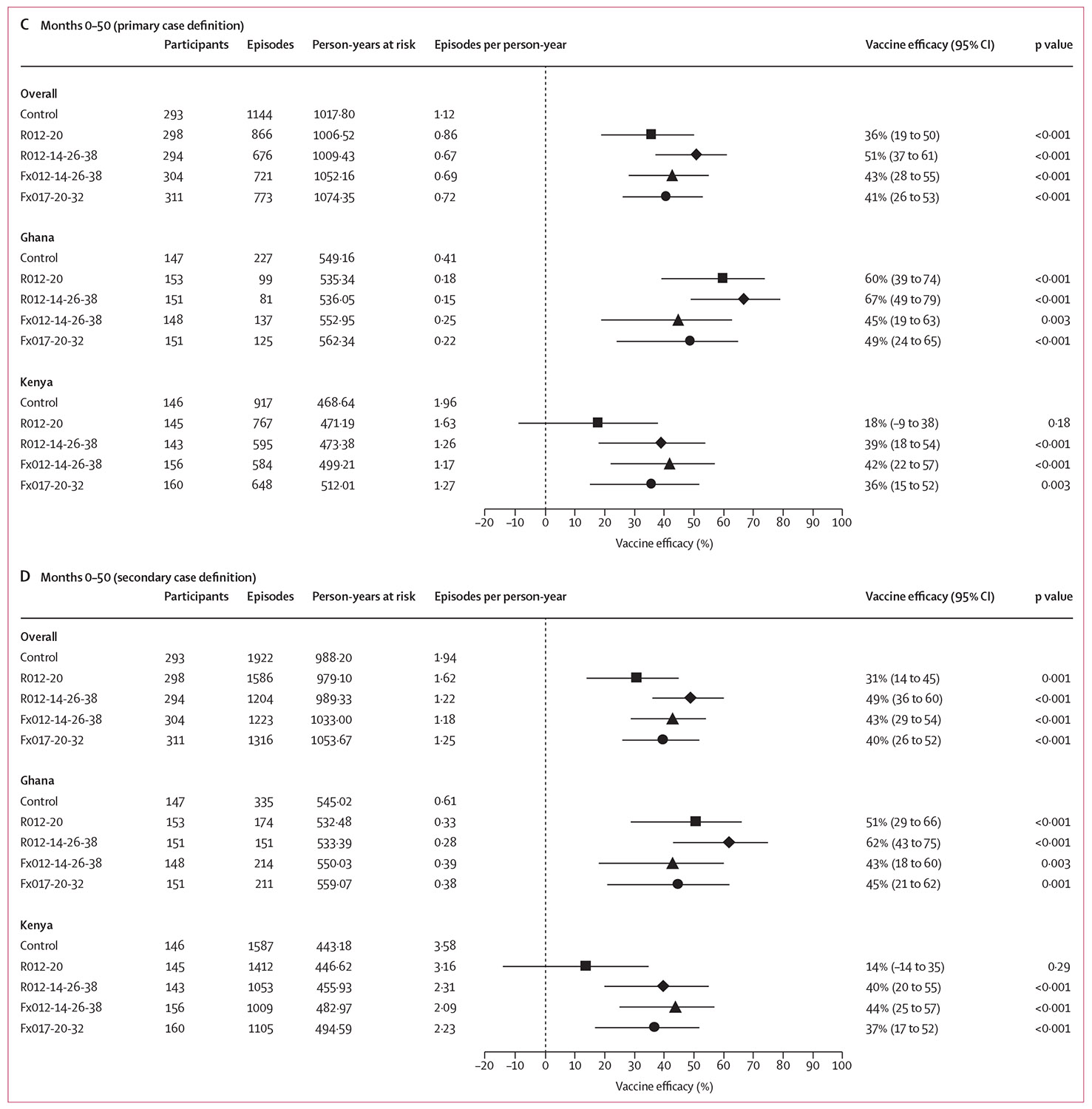
Vaccine efficacy against all episodes of clinical malaria according to the primary (A, C) and secondary (B, D) case definition up to study month 38 (A, B) and 50 (C, D), overall and by country (exposed set) The primary case definition for clinical malaria was *P falciparum* asexual parasitaemia >5000 parasites per μL and fever (axillary temperature ≥37·5°C). The secondary case definition was *P falciparum* asexual parasitaemia >0 parasites per μL and fever or history of fever within 24 h of presentation. Parasitaemia was determined using microscopy.

Overall, in the exposed set, vaccine efficacy over 38 and 50 months of follow-up remained stable and was 38–54% and 31–51%, respectively, regardless of case definition. Point estimates of vaccine efficacy against clinical malaria according to the primary case definition, over 50 months of follow up, were 18% (95% CI −9 to 38) in the R012-20 group versus 39% (18 to 54), 42% (22 to 57), and 36% (15 to 52) in the R012-14-26-38, Fx012-14-26-38, and Fx017-20-32 groups, respectively, in Kenya, and 60% (39 to 74), 67% (49 to 79), 45% (19 to 63), and 49% (24 to 65) in the R012-20, R012-14-26-38, Fx012-14-26-38, and Fx017-20-32 groups, respectively, in Ghana (figure 2). Across RTS,S groups with multiple additional yearly vaccine doses, vaccine efficacy against all episodes of clinical malaria meeting the primary case definition varied from 45% (1–56) in the Fx012-14-26-38 group to 54% (43–64) in the R012-14-26-38 group over 38 months from first dose (months 0–38; figure 2A), and from 41% (26–53) in the Fx017-20-32 group to 51% (37–61) in the R012-14-26-38 group over 50 months from first dose (months 0–50; figure 2C).

In the per-protocol set for efficacy, vaccine efficacy estimates over 12 months of follow-up post-dose 4 were in similar ranges for RTS,S groups. Vaccine efficacy (primary case definition) was 53% (95% CI 34–66) and 57% (39–69) in the Fx012-14-26-38 and R012-14-26-38 groups, respectively, over 12 months of follow-up post-dose 5 and 50% (28–65) in the Fx012-14-26-38 group versus 34% (7–54) in the R012-14-26-38 group, over 12 months of follow-up post-dose 6 (appendix 1 pp 6–8).

When comparing Fx012-14-26-38 with the R012-14-26-38 regimen, in the exposed set, incremental vaccine efficacy against all episodes of clinical malaria over the month 0–50 period was −15% (95% CI −49 to 11; p=0·29) for the primary and −11% (−42 to 13; p=0·39) for the secondary case definition. Incremental vaccine efficacy of the Fx012-14-26-38 versus the R012-20 regimen was also not statistically different: 12% (−14 to 31; p=0·34) for the primary and 18% (−3 to 35; p=0·089) for the secondary case definition.

When assessed by age group, vaccine efficacy in RTS,S groups versus the control group remained statistically significant in children aged 5–8 months at first vaccination but not in the 9–12 months and ≥13 months age groups, regardless of the case definition. In children aged 5–8 months, vaccine efficacy against all episodes of clinical malaria according to the primary case definition ranged from 49% (95% CI 25–66) in the R012-20 group to 66% (48–78) in the Fx012-14-26-38 group (table 1). Vaccine efficacy was also statistically significant in participants with baseline negative parasitaemia but not in those with positive parasitaemia; however, the number of baseline-parasitaemic children in each group was relatively low (appendix 1 p 9).

**Figure 3:**
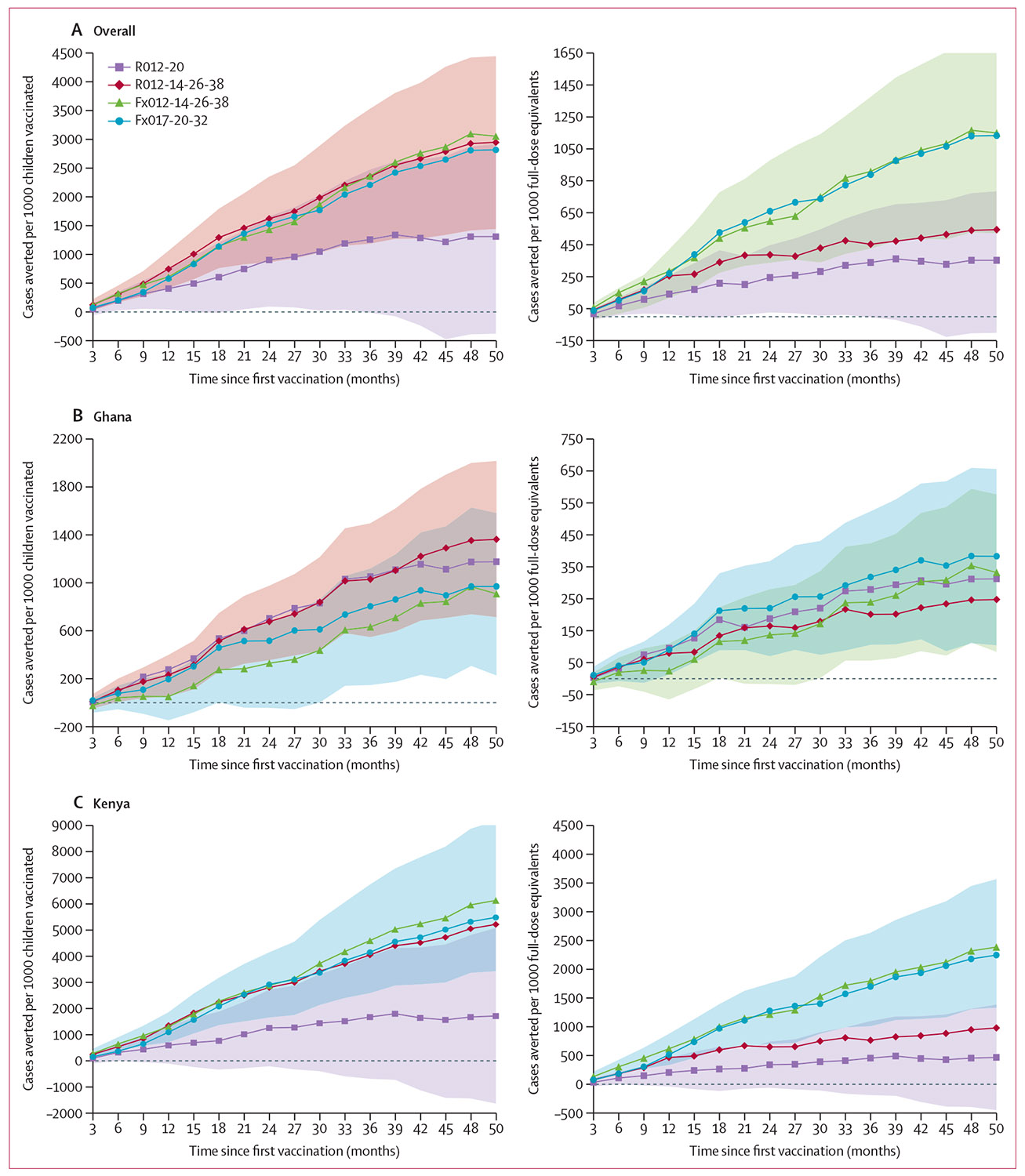
Vaccine impact expressed as cumulative cases averted of clinical malaria (all episodes, secondary case definition) per 1000 children vaccinated or per 1000 full-dose equivalents, overall (A) and by country (B, C) (exposed set) The coloured areas represent the 95% CIs with the highest upper limit and lowest lower limit, respectively, among the four estimates for RTS,S groups.

Overall, 1311 (R012-20 group), 2950 (R012-14-26-38 group), 3054 (Fx012-14-26-38 group), and 2819 (Fx017-20-32 group) cases of clinical malaria (all episodes; secondary case definition) were averted per 1000 children vaccinated compared with the control group, over 50 months of follow-up. The numbers of cases averted per 1000 RTS,S full-dose equivalents were 353 (R012-20 group), 544 (R012-14-26-38 group), 1151 (Fx012-14-26-38 group), and 1134 (Fx017-20-32 group; figure 3, appendix 1 pp 10–11).

The number of cases averted plateaued after month 38 in the R012-20 group, but continued to increase in groups receiving additional yearly doses. This effect was more pronounced in Kenya (figure 3C) than in Ghana (figure 3B). When estimating the effect of the annual vaccine doses, the numbers of cases averted per 1000 children vaccinated over the month 21–50 period were 565 in the R012-20 group and 1491, 1758, and 1454 in the R012-14-26-38, Fx012-14-26-38, and Fx017-20-32 groups, respectively.

Children in RTS,S groups experienced fewer clinical malaria cases than those in the control group, as shown by the distribution of total number of episodes. Up to month 50, 43–49% of children in RTS,S groups versus 32% of children in the control group had no episodes of clinical malaria according to the primary case definition. The proportion of children experiencing more than three episodes increased up to month 50 in all groups, overall and by country, in particular in Kenya. Thus, overall, up to month 38 and month 50, 17–21% and 22–28% of children in the RTS,S groups versus 34% and 37% in the control group experienced more than three episodes of clinical malaria. In Kenya, up to month 50, 54–67% of children in RTS,S groups versus 69% in the control group experienced more than three episodes of clinical malaria (appendix 1 pp 12–13).

The prevalence of *P falciparum* infections was similar across groups. Across all timepoints, values in the control group were 2·5–28·3 times higher in Kenya than in Ghana (appendix 1 p 3). Almost all malaria cases were due to *P falciparum* infection, with very few cases attributed to *P malariae* (<2% of cases) and *P ovale* (<2% of cases); there were no *P vivax* cases.

All RTS,S regimens continued to be immunogenic up to month 50. Anti-circumsporozoite antibody responses peaked after the primary schedule and followed a normal antibody decay curve until each subsequent yearly vaccine dose, with increases observed after each additional dose. Overall, the mean anti-circumsporozoite antibody avidity index increased after each RTS,S dose, including after fractional doses. Anti-hepatitis B surface antibody responses increased after each RTS,S administration, including the additional yearly doses, and remained high and similar between RTS,S groups throughout the study, although they seemed to plateau after dose 4 (appendix 1 pp 4–5).

Solicited adverse events post-doses 3, 4, and 5 and unsolicited adverse events within 30 days from any vaccination occurring up to month 33 were previously reported.^6,7^ Overall, within 4 days after each vaccination (excluding the first two doses), pain was the most common solicited local adverse event, reported after 1–5% of doses in the RTS,S groups but not reported in the control group. Fever was the most frequent general adverse event, reported after 7–20% of doses in the RTS,S groups and 2% in the control group (table 2). The incidence of solicited adverse events after dose 6 was low and similar between groups R012-14-26-38 and Fx012-14-26-38. No grade 3 adverse events were reported. There was no trend of increasing solicited adverse event frequency with each subsequent RTS,S dose.

There was no apparent increase in the frequency of unsolicited adverse events following subsequent RTS,S doses (appendix 1 p 14). Upper respiratory tract infections and malaria were the most commonly reported unsolicited adverse events after each dose. Unsolicited adverse events were reported within 30 days for 64 (28%) of 231 children receiving dose 6 in group R012-14-26-38 and 76 (31%) of 245 children receiving dose 6 in group Fx012-14-26-38. No grade 3 unsolicited adverse events were reported. Causally related adverse events were rare, occurring in seven (3%) of 231 participants in the R012-14-26-38 group and in one (0·4%) of 245 participants in the Fx012-14-26-38 group.

Over 50 months of follow-up, at least one serious adverse event was reported for 21–27% of children in the RTS,S groups and 31·4% of children in the control group (table 2). Up to month 50, eight deaths were reported: two (drowning and malaria) in the R012-20 group, four (gastroenteritis, trauma, thermal burn, and abdominal pain) in the Fx017-20-32 group, and one in each of the R012-14-26-38 (gastroenteritis) and control (malaria) groups; none of these were considered to be related to vaccination. The percentage of children with adverse events of special interest continued to remain low and similar between all groups (table 2).

Cases of aetiology-confirmed meningitis were reported for nine participants. All cases (one in each of the R012-20, R012-14-26-38, and Fx012-14-26-38 groups, three in each of the Fx017-20-32 and control groups) were due to viral meningitis—specifically, herpes simplex virus (one case), Epstein–Barr virus, enterovirus (two cases each), and human beta-herpesvirus 7 (four cases). None were considered related to vaccination.

Severe malaria was reported in 29 (10%) participants in the R012-20 group, 22 (8%) in the R012-14-26-38 group, 25 (8%) in the Fx012-14-26-38 group, 31 (10%) in the Fx017-20-32 group, and 51 (17%) in the control group. One case of cerebral malaria was reported in a participant (0·3%) in the control group. None of these cases of malaria were considered to be related to vaccination.

## Discussion

This is the first study to evaluate fractional-dose regimens of RTS,S in natural exposure settings. Over the 50-month study period, RTS,S demonstrated substantial efficacy of all four vaccine regimens compared with the control, in line with observations at month 21^6^ and month 32.^7^ RTS,S was previously shown to provide protection against malaria and severe malaria up to 7 years from administration of a four-dose schedule.^15^

Overall, after longer follow-up periods in our study, both vaccine efficacy and vaccine impact were non-statistically significantly lower in the standard full-dose R012-20 group compared with the other RTS,S groups that received additional yearly doses. To show that this trend was not an artefact of the unexplained lower efficacy and impact noted in the R012-20 group from as early as month 14, we subtracted cumulative averted cases of clinical malaria up to month 21 from the number of cases averted at month 50 in each group. We still observed that 2–3 times more clinical malaria cases were averted in groups receiving five or six doses compared with the R012-20 group receiving only four doses, suggesting a benefit from yearly doses beyond the fourth dose. We also observed that point estimates of vaccine efficacy and the number of cases averted tended to be higher in both the full-dose and fractional-dose groups who received three additional yearly RTS,S doses than in the Fx017-20-32 group with only two additional yearly vaccine doses, although the difference was not statistically significant. Additionally, a higher number of clinical malaria cases seemed to be averted when using the Fx012-14-26-38 regimen compared with all other RTS,S regimens evaluated, although the statistical significance of this difference was not assessed. This effect was more pronounced for the Kenyan site, which had a considerably higher prevalence of *P falciparum* infections compared with the Ghanaian site throughout the entire study period. Therefore, our findings suggest that multiple yearly, fractional RTS,S doses are beneficial in maintaining protection against clinical malaria even after 4 years post-first vaccination, especially in areas of high malaria endemicity.

Vaccine efficacy was similar between fractional-dose and full-dose RTS,S regimens. By factoring in the volume of vaccine administered, we found that vaccine impact point estimates for fractional-dose regimens tended to be higher than that for the full-dose regimens, with the difference in cases of clinical malaria averted increasing from month 21 to month 50, and indicating a potential dose-sparing effect. These differences between fractional-dose and full-dose regimens were more pronounced in Kenya than in Ghana, but were not statistically significant. When considering only the cases averted following administration of additional yearly doses, vaccine impact was still higher in groups Fx012-14-26-38 and Fx017-20-32 than in the full-dose groups. Therefore, the impact of additional yearly fractional vaccine doses appeared to be higher than that of yearly full-dose vaccinations, particularly in Kenya. Although this study was not designed to assess non-inferiority between vaccine regimens, our findings indicate that multiple additional yearly vaccinations can be administered as fractional doses without diminishing the vaccine efficacy, especially in areas with high *P falciparum* prevalence. We also found that vaccine efficacy and impact were within similar ranges in the Fx012-14-26-38 and Fx017-20-32 groups, suggesting a potential flexibility in the timing of primary RTS,S vaccinations, as well as for additional yearly, fractional vaccine doses.

For all RTS,S groups, over the 50-month follow-up period, vaccine efficacy against all episodes of clinical malaria was higher in children aged 5–8 months than in those aged 9–12 or ≥13 months at first vaccination, regardless of the case definition used. Vaccine efficacy point estimates also tended to be higher in children with negative parasitaemia compared with those positive at baseline (except for group Fx017-20-32, based on the secondary case definition). However, the number of children with parasitaemia at baseline was low and the statistical significance of this difference was not assessed; therefore, these results should be interpreted with caution. Nevertheless, this finding seems to indicate that early RTS,S administration, when the child is at lower risk of being infected at the time of the first vaccination, could lead to better protection against disease. In a study that evaluated the effect of malaria infection on RTS,S vaccine efficacy in children aged 5–17 months at first vaccination,^16^ the authors found that vaccine efficacy was not related to infections during vaccination and suggested that control group immunity rather than infections before or during vaccination could be the reason for lower efficacy in high-transmission settings.^16^ In our study, we also observed a tendency towards higher point estimates of RTS,S vaccine efficacy in lower (Ghana) than in higher (Kenya) transmission settings after three doses, although this difference was not statistically significant and was no longer observed after yearly fractional-dose vaccinations. Of note, in an ancillary genotyping study using pooled samples for RTS,S groups up to month 20, it was found that vaccine efficacy against a first new infection (genotypically detected) was significantly higher in children who were malaria-infected (68%) versus uninfected (37%) at the first vaccination.^17^ However, any comparison with the genotyping study is hindered by the fact that vaccine efficacy was evaluated against clinical disease in the current study, whereas genotyping is a more sensitive assay and might have allowed the identification of more baseline parasite-positive children, thus increasing the power to observe differences. Additionally, one could hypothesise that baseline-infected individuals had pre-existing infections that subsequently converted to symptomatic cases during the follow-up period, which would detract from any added protective effect of baseline infection on subsequent clinical disease. In high-transmission settings, many infections are below the level of detection by microscopy and individuals are exposed to multiple simultaneously occurring infections (multiplicity or complexity of infection and superinfections) that cannot be detected by microscopy. Therefore, our ability to measure vaccine efficacy, assuming a vaccine at least partially prevents some of these events, is disproportionately limited, and likely underestimates vaccine efficacy in high-transmission settings. Thus, although a protective effect of baseline infections could be established against subsequent infection when using genotyping, it is difficult to make a similar inference about clinical disease given that infections were not drug-cleared before the first vaccination.

In our study, throughout 50 months of follow-up, children continued to experience malaria cases, including multiple episodes for the same child. At the Kenyan site, more than 54% of children in the control group had experienced more than three episodes of clinical malaria (regardless of the case definition), underscoring the importance of repeated annual vaccinations, at least up to 5 years of age. However, there were fewer cases of severe malaria in RTS,S groups than in the control group, suggesting no evidence of rebound of severe malaria, in line with previous findings.^15^ We found that the timing of multiple additional yearly doses did not greatly impact efficacy of RTS,S regimens, enhancing the adaptability of these regimens for successful integration into the Expanded Programme on Immunization. Taken together, our results suggest that RTS,S fractional-dose regimens including multiple additional yearly fractional doses can be successfully used to prevent clinical malaria. This could reduce the costs and increase the availability of the RTS,S vaccination because more children could be vaccinated when using fractional instead of standard full doses.

Immune responses induced by the fractional-dose regimens continued to be similar to those in full-dose groups, as observed at month 21^6^ and month 33.^7^ In all RTS,S groups, a trend for a decline in anti-circumsporozoite antibody geometric mean concentration point estimates was observed after subsequent yearly doses, but 95% CIs were overlapping, in line with previous findings.^18,19^ However, this trend did not translate into a significant effect on vaccine efficacy in either group.

All RTS,S regimens continued to be well tolerated, with the incidence of solicited and unsolicited adverse events similar to or lower than those observed in the phase 3 trial in African children aged 5–17 months.^20^ Multiple doses did not seem to lead to an increase in the frequency of adverse events, and no concerning pattern of harms was identified for regimens with either fractional or full additional yearly vaccine doses. Throughout the study, severe malaria cases were reported in up to 10% of children in RTS,S groups and 17% of children receiving the control vaccine, and no cases were considered related to RTS,S vaccination. There was no indication of an increased risk for meningitis or cerebral malaria in the RTS,S groups compared with the control group, similar to observations from the Malaria Vaccine Implementation Programme.^2,21^ This harm profile is further reinforced by adverse event findings after the administration of more than 6 million doses of RTS,S in the Malaria Vaccine Implementation Programme.^22^

Our study is not without limitations, including the open-label design. Any group comparisons should be interpreted with caution since the primary objective could not be demonstrated (objectives were assessed in an hierarchical manner) and due to the multiplicity of objectives. In addition, comparisons by country are limited by the fact that the study was not powered to assess efficacy by site and the known difference in malaria transmission for the two sites. Further studies could investigate the optimisation of fractional dosing of the vaccine adjuvant and antigen.

In conclusion, all fractional-dose and full-dose RTS,S regimens were well tolerated, immunogenic, and provided substantial protection against malaria over 50 months of follow-up. Our results indicate that RTS,S dose frequency might be more important than the amount of vaccine antigen administered in maintaining vaccine efficacy over time and support the administration of multiple full or fractional additional yearly doses up to 5 years of age. The use of fractional doses would increase the number of children who can be vaccinated with the available RTS,S doses and reduce the cost of vaccination without an effect on protection against malaria. This can be further investigated in real-life settings, in the context of the wide implementation of RTS,S vaccination.

## Supplementary Material

Supplementary Appendix 2

Supplementary Appendix 1

## Figures and Tables

**Table 1:** Vaccine efficacy against all episodes of clinical malaria up to month 50 in participants aged 5–8, 9–12, and ≥13 months at first vaccination (exposed set)

	Participants*	Episodes†	Person-years at risk	Person-year rate‡	Vaccine efficacy (95% CI)	p value§
**Age 5–8 months**						
Primary case definition						
Control	110	407	391·23	1·04	··	··
R012-20	126	285	430·39	0·66	49% (25 to 66)	<0·001
R012-14-26-38	125	270	436·88	0·62	60% (41 to 73)	<0·001
Fx012-14-26-38	119	195	423·22	0·46	66% (48 to 78)	<0·001
Fx017-20-32	135	297	484·20	0·61	51% (29 to 66)	<0·001
Secondary case definition						
Control	110	629	382·84	1·64	··	··
R012-20	126	518	421·54	1·23	42% (16 to 60)	0·004
R012-14-26-38	125	473	429·18	1·10	53% (33 to 67)	<0·001
Fx012-14-26-38	119	344	417·51	0·82	60% (41 to 72)	<0·001
Fx017-20-32	135	512	475·99	1·08	45% (22 to 60)	<0·001
**Age 9–12 months**						
Primary case definition						
Control	75	335	252·30	1·33	··	··
R012-20	80	298	262·24	1·14	22% (−17 to 48)	0·23
R012-14-26-38	77	245	262·10	0·93	38% (4 to 60)	0·031
Fx012-14-26-38	77	218	269·40	0·81	40% (8 to 61)	0·019
Fx017-20-32	76	218	255·41	0·85	47% (14 to 67)	0·009
Secondary case definition						
Control	75	573	243·20	2·36	··	··
R012-20	80	536	253·17	2·12	27% (−12 to 52)	0·15
R012-14-26-38	77	412	255·74	1·61	43% (13 to 63)	0·010
Fx012-14-26-38	77	355	264·15	1·34	45% (19 to 63)	0·003
Fx017-20-32	76	392	248·82	1·58	44% (15 to 63)	0·008
**Age ≥13 months**						
Primary case definition						
Control	108	402	374·27	1·07	··	··
R012-20	92	283	313·89	0·90	31% (−7 to 56)	0·097
R012-14-26-38	92	161	310·44	0·52	51% (22 to 69)	0·003
Fx012-14-26-38	108	308	359·55	0·86	14% (−26 to 41)	0·44
Fx017-20-32	100	258	334·74	0·77	20% (−19 to 46)	0·27
Secondary case definition						
Control	108	720	362·17	1·99	··	··
R012-20	92	532	304·39	1·75	20% (−19 to 46)	0·28
R012-14-26-38	92	319	304·41	1·05	51% (22 to 69)	0·003
Fx012-14-26-38	108	524	351·34	1·49	20% (−17 to 45)	0·25
Fx017-20-32	100	412	328·86	1·25	28% (−5 to 51)	0·089

* Number of children in each group contributing to the considered evaluation period.

† Number of episodes included in each group.

‡ Calculated as: number of episodes/person-years at risk.

§ The p values were calculated using a negative binomial model. Since the primary objective of the study was not demonstrated, any group comparisons should be interpreted with caution.

**Table 2:** Summary of adverse events

	R012-20	R012-14-26-38	Fx012-14-26-38	Fx017-20-32	Control
**Solicited adverse events over the 4-day follow-up period following each vaccination starting from dose 3 up to last vaccine dose (reactogenicity subset)**					
Number of doses received	87	173	163	131	49
Local adverse events					
Erythema	0 (0%; 0–4)	1 (1%; 0–3)	0 (0%; 0–2)	0 (0%; 0–3)	0 (0%; 0–7)
Pain	2 (2%; 0–8)	9 (5%; 2–10)	1 (1%; 0–3)	4 (3%; 1–8)	0 (0%; 0–7)
Medically attended	1 (1%; 0–6)	3 (2%; 0–5)	0 (0%; 0–2)	1 (1%; 0–4)	0 (0%; 0–7)
Swelling	1 (1%; 0–6)	1 (1%; 0–3)	1 (1%; 0–3)	3 (2%; 0–7)	0 (0%; 0–7)
Medically attended	1 (1%; 0–6)	1 (1%; 0–3)	0 (0%; 0–2)	0 (0%; 0–3)	0 (0%; 0–7)
General adverse events					
Drowsiness	2 (2%; 0–8)	2 (1%; 0–4)	3 (2%; 0–5)	2 (2%; 0–5)	0 (0%; 0–7)
Related	1 (1%; 0–6)	0 (0%; 0–2)	2 (1%; 0–4)	1 (1%; 0–4)	0 (0%; 0–7)
Medically attended	1 (1%; 0–6)	1 (1%; 0–3)	2 (1%; 0–4)	1 (1%; 0–4)	0 (0%; 0–7)
Irritability or fussiness	0 (0%; 0–4)	7 (4%; 2–8)	0 (0%; 0–2)	4 (3%; 1–8)	0 (0%; 0–7)
Related	0 (0%; 0–4)	3 (2%; 0–5)	0 (0%; 0–2)	2 (2%; 0–5)	0 (0%; 0–7)
Medically attended	0 (0%; 0–4)	6 (3%; 1–7)	0 (0%; 0–2)	2 (2%; 0–5)	0 (0%; 0–7)
Loss of appetite	2 (2%; 0–8)	6 (3%; 1–7)	3 (2%; 0–5)	1 (1%; 0–4)	0 (0%; 0–7)
Related	1 (1%; 0–6)	1 (1%; 0–3)	2 (1%; 0–4)	0 (0%; 0–3)	0 (0%; 0–7)
Medically attended	2 (2%; 0–8)	5 (3%; 1–7)	2 (1%; 0–4)	1 (1%; 0–4)	0 (0%; 0–7)
Fever	17 (20%; 12–29)	31 (18%; 13–24)	11 (7%; 3–12)	10 (8%; 4–14)	1 (2%; 0–11)
Grade 3	2 (2%; 0–8)	1 (1%; 0–3)	2 (1%; 0–4)	0 (0%; 0–3)	0 (0%; 0–7)
Related	13 (15%; 8–24)	20 (12%; 7–17)	7 (4%; 2–9)	5 (4%; 1–9)	0 (0%; 0–7)
Grade 3 related	2 (2%; 0–8)	1 (1%; 0–3)	0 (0%; 0–2)	0 (0%; 0–3)	0 (0%; 0–7)
Medically attended	8 (9%; 4–17)	17 (10%; 6–15)	6 (4%; 1–8)	4 (3%; 1–8)	1 (2%; 0–11)
**Serious adverse events and adverse events of special interest reported up to study end (exposed set)**					
Number of children	298	294	304	311	293
Serious adverse events	73 (25%; 20–30)	65 (22%; 18–27)	64 (21%; 17–26)	84 (27%; 22–32)	92 (31%; 26–37)
Related serious adverse events	3 (1%; 0–3)	0 (0%; 0–1)	0 (0%; 0–1)	2 (0·6%; 0–2)	0 (0%; 0–1)
Adverse events of special interest					
Meningitis	1 (0·3%; 0–2)	1 (0·3%; 0–2)	1 (0·3%; 0–2)	3 (1%; 0–3)	3 (1%; 0–3)
Seizure within 30 days post-vaccination	12 (4%; 2–7)	6 (2%; 0–4)	6 (2%; 1–4)	13 (4%; 2–7)	12 (4%; 2–7)
pIMD	0 (0%; 0–1)	2 (0·7%; 0–2)	1 (0·3%; 0–2)	0 (0%; 0–1)	0 (0%; 0–1)
Cerebral malaria	0 (0%; 0–1)	0 (0%; 0–1)	0 (0%; 0–1)	0 (0%; 0–1)	1 (0·3%; 0–2)
Severe malaria	29 (10%; 7–14)	22 (8%; 5–11)	25 (8%; 5–12)	31 (10%; 7–14)	51 (17%; 13–22)
Deaths*	2 (0·7%; 0–2)	1 (0·3%; 0–2)	0 (0%; 0–1)	4 (1%; 0–3)	1 (0·3%; 0–2)

Data are n (%; 95% CI) unless otherwise indicated. n (%) refers to number (percentage) of doses followed by least one adverse event for solicited adverse events, and number (percentage) of children with at least one adverse event for serious adverse events and adverse events of special interest. For solicited adverse events, the analysis included all children within the reactogenicity subset who had safety data; all solicited local adverse events were considered related to vaccination. Grade 3 solicited adverse events were defined as erythema or swelling >20 mm, crying when limb is moved (pain), not eating at all (loss of appetite), preventing normal everyday activities (drowsiness or irritability or fussiness), and temperature >39·0°C (fever). For serious adverse events and adverse events of special interest, the analysis included all children who had safety data. Exact 95% CIs were calculated using the Clopper–Pearson method. pIMD=potential immune-mediated disease. *Deaths were as follows (ages at enrolment are provided): malaria of moderate severity in an 11-month-old girl with onset at 221 days after the fourth dose (blood slide was positive for malaria parasites and symptoms were body temperature of 40°C and vomiting), a case of drowning in a 16-month-old boy at 251 days after the third dose (R012-20 group); a case of severe gastroenteritis in a 15-month-old girl with onset at 204 days after the third dose (R012-14-26-38 group); a case of severe gastroenteritis in an 11-month-old girl with onset at 228 days after the third dose, thermal burns in a 14-month-old girl at 100 days after the fourth dose, abdominal pain in a 14-month-old boy with onset at 78 days after the fourth dose, trauma from a wall accidentally falling on a 6-month-old boy at 270 days after the third dose (Fx017-20-32 group); and severe malaria in a 7-month-old boy with onset at 770 days after receiving the third dose of rabies vaccine (control group).

## Data Availability

Anonymised individual participant data and study documents will be available at study end, when they can be requested for further research from www.clinicalstudydatarequest.com (study ID: 204889).

## Research in context

### Evidence before this study

We conducted a systematic literature search of PubMed on March 14, 2024, for publications reporting results of clinical trials of the RTS,S/AS01 malaria vaccine using a fractional dose.

We used the search string ((“RTS,S” [All Fields]) AND (“fractional” [All Fields])). Of the 13 manuscripts identified, ten reported results of clinical trials: four reported vaccine efficacy results from controlled human malaria infection (CHMI) studies in adults and four further investigated immune responses using data from these CHMI studies. The other two publications reported intermediate results over 21 and 32 months of follow-up of the current study (NCT03276962), including the results for the primary objective.

### Added value of this study

This is the first study to evaluate a fractional-dose regimen of RTS,S/AS01_E_ in children aged 5–17 months, in natural exposure settings in countries with moderate-to-high malaria transmission. We evaluated different vaccination regimens using full doses at months 0 and 1 and either full dose (R) or one-fifth of a full dose (fractional dose; Fx) as subsequent doses, administered according to different schedules: R012-20, R012-14-26-38, Fx012-14-26-38, and Fx017-20-32. We did not find superiority of the Fx012 versus the R012 regimen over 12 months of follow-up post-dose 3 (primary objective).

However, throughout 50 months of follow-up, vaccine efficacy against all episodes of clinical malaria (calculated as 100 × [1 – incidence rate ratio]) and vaccine impact (expressed as cumulative cases averted of clinical malaria) for groups R012-14-26-38, Fx012-14-26-38, and Fx017-20-32, receiving yearly vaccinations after the fourth dose, tended to be higher than those in the R012-20 group. When accounting for the volume of vaccine administered, the number of clinical malaria cases averted tended to be higher in the fractional-dose groups than in the full-dose groups. Administration of multiple yearly doses led to sustained vaccine efficacy and impact over 50 months of follow-up. All regimens were immunogenic and well tolerated.

### Implications of all the available evidence

Our results indicate that RTS,S/AS01_E_ regimens including multiple yearly vaccine doses according to different schedules lead to sustained protection against clinical malaria up to 5 years of age. We found that additional yearly vaccine doses can be administered as fractional doses and with a certain flexibility in the timing of vaccination, leading to similar vaccine efficacy and impact compared with full-dose regimens. Using fractional regimens of RTS,S/AS01_E_ could increase vaccine availability and reduce the cost of vaccination without reducing protection against malaria.
