# Supplementary material for: Sustained efficacy of the RTS,S/AS01E malaria vaccine over 50 months of follow-up when used in full-dose or fractional-dose regimens in young children in Ghana and Kenya: final results from an open-label, phase 2b, randomised controlled trial
Source: Lancet Glob Health. Author manuscript; Available in PMC 2026 May 11. (PMC13159482; doi:10.1016/S2214-109X(25)00272-4)
Supplement: Supplementary Appendix 2 [file NIHMS2166155-supplement-Supplementary_Appendix_2.pdf]

THE LANCET
Global Health

Supplementary appendix 2

This Equitable Partnership Declaration (EPD) was submitted by the authors, and we reproduce it as supplied. It has not been peer reviewed. *The Lancet*'s editorial processes have not been applied to the EPD.

Supplement to: Osei-Tutu L, Kariuki SK, Lee CK, et al. Sustained efficacy of the RTS,S/AS01$_E$ malaria vaccine over 50 months of follow-up when used in full-dose or fractional-dose regimens in young children in Ghana and Kenya: final results from an open-label, phase 2b, randomised controlled trial. *Lancet Glob Health* 2025; **13:** e1723–36.

Equitable Partnership Declaration questions

Researcher considerations

1. Please detail the involvement that researchers who are based in the region(s) of study had during a) study design; b) clinical study processes, such as processing blood samples, prescribing medication, or patient recruitment; c) data interpretation; and d) manuscript preparation, commenting on all aspects. If they were not involved in any of these aspects, please explain why.

   This question is intended for international partnerships; if all your authors are based in the area of study, this question is not applicable.

   This should include a thorough description of their leadership role(s) in the study. Are local researchers named in the author list or the acknowledgements, or are they not mentioned at all (and, if not, why)? Please also describe the involvement of early career researchers based in the location of the study. Some of this information might be repeated from the Contributors section in the manuscript. Note: we adhere to ICMJE authorship criteria when deciding who should be named on a paper.

**a) Study design:**<br>Simon K Kariuki Martina Oneko, Nelli Westercamp, Daniel Ansong, Tsiri Agbenyega, Aaron M. Samuels were involved in the conception or the design of the study
**b) Clinical study processes:**<br>Lawrence Osei-Tutu, Simon K Kariuki, Dennis K Bii, Samuel Adjei, Martina Oneko, Maame Anima Attobrah Sarfo, Patrick Boakye Yiadom Buabeng, Ashura Bakari, Cecilia Atieno, Maame Fremah Kotoh-Mortty, Kephas Otieno, Yaw Ntiamoah, Tony Sang, Daniel Ansong, Tsiri Agbenyega, Aaron M. Samuels collected the study data as site investigators.
**c) Data interpretation:**<br>Lawrence Osei-Tutu, Simon K Kariuki, Dennis K Bii, Samuel Adjei, Martina Oneko, Patrick Boakye Yiadom Buabeng, Daniel Ansong, Tsiri Agbenyega, Aaron M. Samuels interpreted the data as site investigators.
**d) Manuscript preparation:**<br>Lawrence Osei-Tutu, Simon K Kariuki, Dennis K Bii, Samuel Adjei, Martina Oneko, Maame Anima Attobrah Sarfo, Patrick Boakye Yiadom Buabeng, Ashura Bakari, Cecilia Atieno, Maame Fremah Kotoh-Mortty, Kephas Otieno, Yaw Ntiamoah, Tony Sang, Daniel Ansong, Tsiri Agbenyega, Aaron M. Samuels reviewed manuscript draft and approved the final version as site investigators.

2. Were the data used in your study collected by authors named on the paper, or have they been extracted from a source such as a national survey? ie, is this a secondary analysis of data that were not collected by the authors of this paper. If the authors of this paper were not involved in data collection, how were data interpreted with sufficient contextual knowledge?

   The Lancet Global Health *believe contextual understanding is crucial for informed data analysis and interpretation.*

The data were collected by authors named on the paper.

3. How was funding used to remunerate and enhance the skills of researchers and institutions based in the area(s) of study? And how was funding used to improve research infrastructure in the area of study?

   Potentially effective investments into long-term skills and opportunities within institutions could include training or mentorship in analytical techniques and manuscript writing, opportunities to lead all or specific aspects of the study, financial remuneration rather than requiring volunteers, and other professional development and educational opportunities.

   Improvements to research infrastructure could be funding of extended trial designs (such as platform trials) and use of master protocols to enable these designs, establishment of long-term contracts for research staff, building research facilities, and local control of funding allocation.

**Skills:**<br>*The health systems in the study areas were improved through training provided by GSK and external partners on lab methodology (handling of samples, shipment of different lab samples used for testing and vaccine management), pharmacovigilance and the diagnosis of Adverse Events of Special Interest (AESIs). Throughout the study there were investigator meetings held where all staff had opportunities to discuss and input into methodology for collecting data, discuss results, request additional analyses. The company trained the laboratory staff and through audits helped to improve operating procedures. The manuscript writing provided opportunities for mid-career scientists to input and learn methodologies through open discussions with more senior colleagues and also allowed all study team members to review and understand how to write and critically assess data produced- all comments during the development of the manuscript were shared openly as well the comments from the journal. These were used as teaching moments through the discussions. Additionally, aids through an external partner were supplied to assist in the correct identification of AESIs, and sampling and laboratory testing capabilities for cerebrospinal fluid analysis were enhanced.*
**Research infrastructure:**<br>*Laboratory equipment was procured for study investigations. The site will continue to help the community where the research centre is located using some of these equipment.*

4. How did you safeguard the researchers who implemented the study?

   Please describe how you guaranteed safe working conditions for study staff, including provision of appropriate personal protective equipment, protection from violence, and prevention of overworking.

We ensured safe working conditions for study staff by providing appropriate personal protective equipment for biological sample handling. All staff members followed good clinical and relevant laboratory practices. Sita assessments by externals paid by the company enhanced operating procedures used for the volunteers, the staff and the laboratories.

Benefits to the communities and regions of study

5. How does the study address the research and policy priorities of its location?

   How were the local priorities determined and then used to inform the research question? Who decided which priorities to take forward? Which elements of the study address those priorities?

Local priorities were determined by the high burden of malaria among children under 5 in sub-Saharan Africa. The study was designed in partnership with the PIs and selected staff. The appropriate methodologies that were useful for the region were discussed, agreed upon and used during the study. The study principal investigators and their staff identified areas/infrastructure needs that were paid by the company to ensure the safety and well-being of volunteers and staff.

6. How will research products be shared in the community of study?

   For instance, will you be providing written or oral layperson summaries for non-academic information sharing? Will study data be made available to institutions in the region(s) of study? The Lancet Global Health *encourages authors to translate the summary (abstract) into relevant languages after paper editing; do you intend to translate your summary?*

The study protocol outline results are publicly available (Home | ClinicalTrials.gov) and Pan African Clinical Trials Registry (PACTR)-
Written layperson summaries were developed by the company and PIs to be distributed to all participants parents/guardians/community leaders. The study staff developed results oral layperson summaries in English and the appropriate languages of the study area which were presented to all the communities where the study took place.

7. How were individuals, communities, and environments protected from harm?

a) How did you ensure that sensitive patient data was handled safely and respectfully? Was there any potential for stigma or discrimination against participants arising from any of the procedures or outcomes of the study?

Sensitive patient data was handled safely and respectfully by ensuring that only researchers at the study sites could access identifiable information, such as names and addresses, solely for the study's purpose. All study information was labelled with a code number instead of personal identifiers, and only the study doctor had the link between the participants' names and the code numbers. Only coded information was sent to GSK.
Additionally, subject data necessary for analysis and reporting was entered/transmitted into a validated data system, ensuring data integrity and security.
Freely given and written or witnessed and thumb-printed informed consent was obtained from each subject's parent(s)/ legally acceptable representative(s) prior to participation in the study, ensuring that participants were fully informed and voluntarily agreed to participate.
The ICFs were also kept under lock and key, only the PI and the program manager had access to it.

b) Might any of the tests be experienced as invasive or culturally insensitive?

No - all process were developed in partnership with the study staff.

c) How did you determine that work was sensitive to traditions, restrictions, and considerations of all cultural and religious groups in the study population?

All process were developed in partnership with the study staff

d) Were biowaste and radioactive waste disposed of in accordance with local laws?

Yes

e) Were any structures built that would have impacted members of the community or the environment (such as handwashing facilities in a public space)? If so, how did you ensure that you had appropriate community buy-in?

No – appropriate infrastructure as needed were built in the study centres and hospitals where data was collected.

f) *How might the study have impacted existing health-care resources (such as staff workloads, use of equipment that is typically employed elsewhere, or reallocation of public funds)?*

The study had assigned rooms, infrastructure and staff paid for to work on the study.

8. Finally, please provide the title (eg, Dr/Prof, Mr/Mrs/Ms/Mx), name, and email address of an author who can be contacted about this statement. This can be the corresponding author.

Name: Dr Opokua Ofori-Anyinam
Email: opokua.ofori-anyinam@gsk.com
