# Supplementary material for: Sustained efficacy of the RTS,S/AS01E malaria vaccine over 50 months of follow-up when used in full-dose or fractional-dose regimens in young children in Ghana and Kenya: final results from an open-label, phase 2b, randomised controlled trial
Source: Lancet Glob Health. Author manuscript; Available in PMC 2026 May 11. (PMC13159482; doi:10.1016/S2214-109X(25)00272-4)
Supplement: Supplementary Appendix 1 [file NIHMS2166155-supplement-Supplementary_Appendix_1.pdf]

THE LANCET
Global Health

Supplementary appendix 1

This appendix formed part of the original submission and has been peer reviewed. We post it as supplied by the authors.

Supplement to: Osei-Tutu L, Kariuki SK, Lee CK, et al. Sustained efficacy of the RTS,S/AS01$_E$ malaria vaccine over 50 months of follow-up when used in full-dose or fractional-dose regimens in young children in Ghana and Kenya: final results from an open-label, phase 2b, randomised controlled trial. *Lancet Glob Health* 2025; **13:** e1723–36.

Appendix

Figure S1. Study design overview

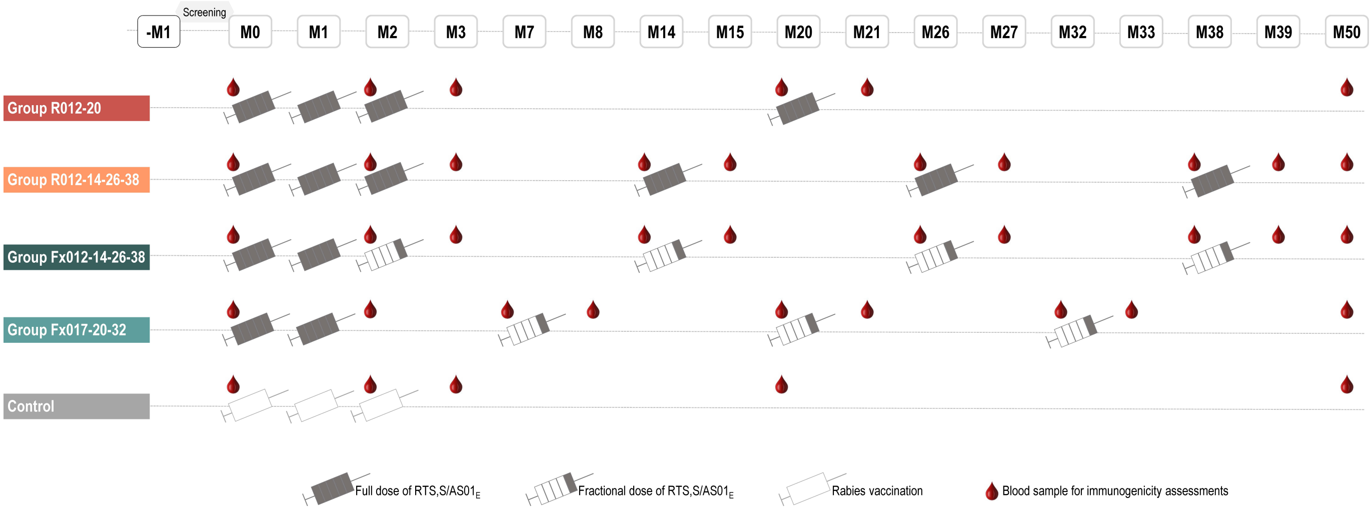

M, month.

Study information

Study group and intervention number allocation

Allocation of the participant to a study group at the investigator site was performed using a randomisation system on internet (SBIR). The randomisation algorithm used a minimization procedure accounting for centre. After obtaining the signed (or thumb-printed and witnessed) and dated informed consent form from the participant's parent(s)/legally acceptable representative(s) and having checked the eligibility of the child, the site staff in charge of the vaccine administration accessed SBIR. Upon providing the participant identification number, the randomisation system determined the study group and provided the intervention number to be used for the first dose. For each subsequent dose, the study staff in charge of the vaccine administration accessed SBIR, provided the participant identification number, and the system provided a treatment number consistent with the allocated study group. The number of each administered treatment was recorded in the electronic case report form for each participant.

Ethics approval

Institutional review boards/independent ethics committees approved the trial protocol: the Committee for Human Research Publication and Ethics at the Kwame Nkrumah University of Science and Technology, the Ghana Health Services Ethics Review Committee, the Ghana Food and Drugs Authority, the Kenya Medical Research Institute Scientific and Ethics Review Unit (SSC #3564), the Western Institutional Review Board, the United States Centers for Disease Control and Prevention Institutional Review Board (Protocol #7058), and the Kenya Pharmacy and Poisons Board.

Figure S2. Prevalence of *Plasmodium falciparum* infections at each cross-sectional visit, from month 1 to month 50, overall (A) and by country (Ghana [B] and Kenya [C]) (exposed set)

A. Overall

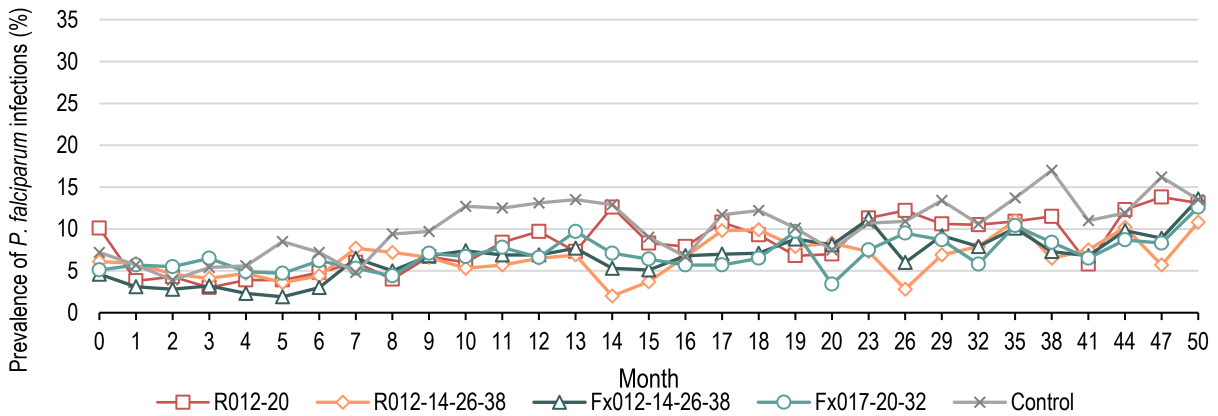

B. Ghana

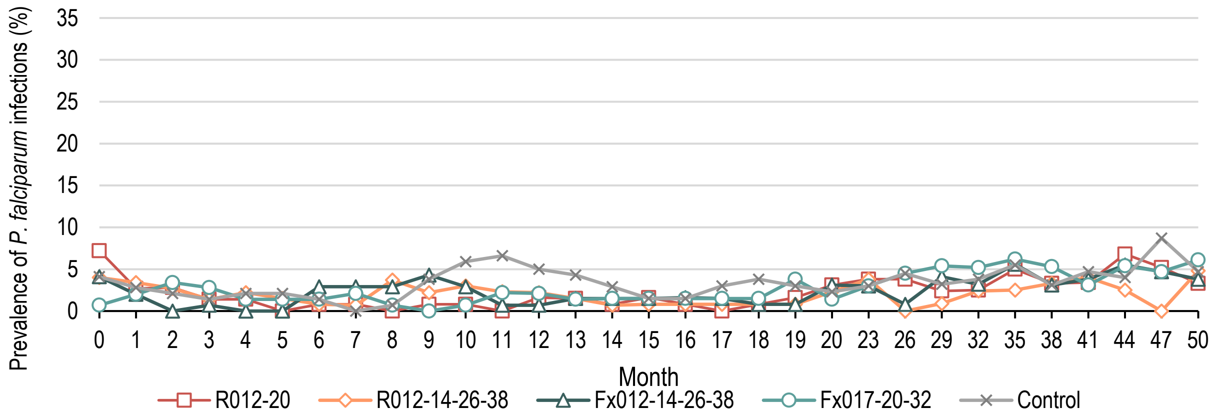

C. Kenya

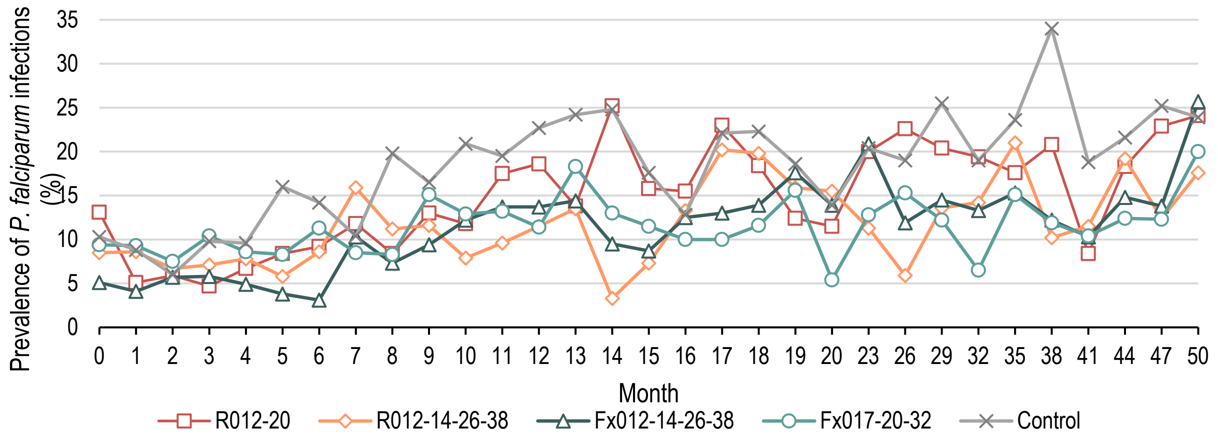

Note: The prevalence of *P. falciparum* infections was assessed at at each cross-sectional visit, i.e., each calendar month up to month 20 and every three months thereafter, up to study end (month 50).

Figure S3. Immune responses to vaccination: anti-CS antibody GMCs (A, A'), anti-CS antibody avidity index (B, B'), and anti-HBs antibody GMCs (C, C'), by timepoint (A, B, C) and by month from first dose (A', B', C') (immunogenicity subset, per-protocol set for immunogenicity)

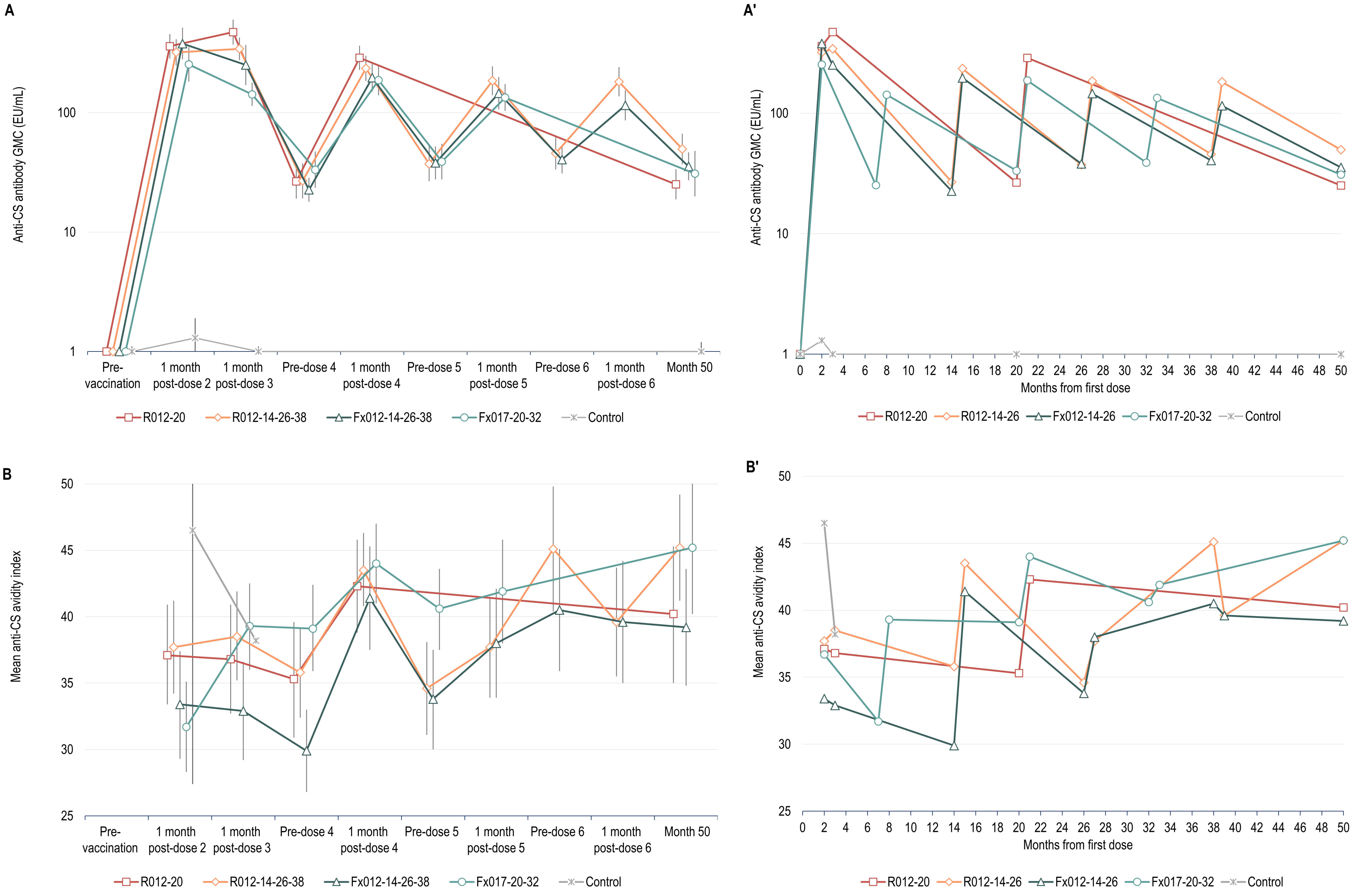

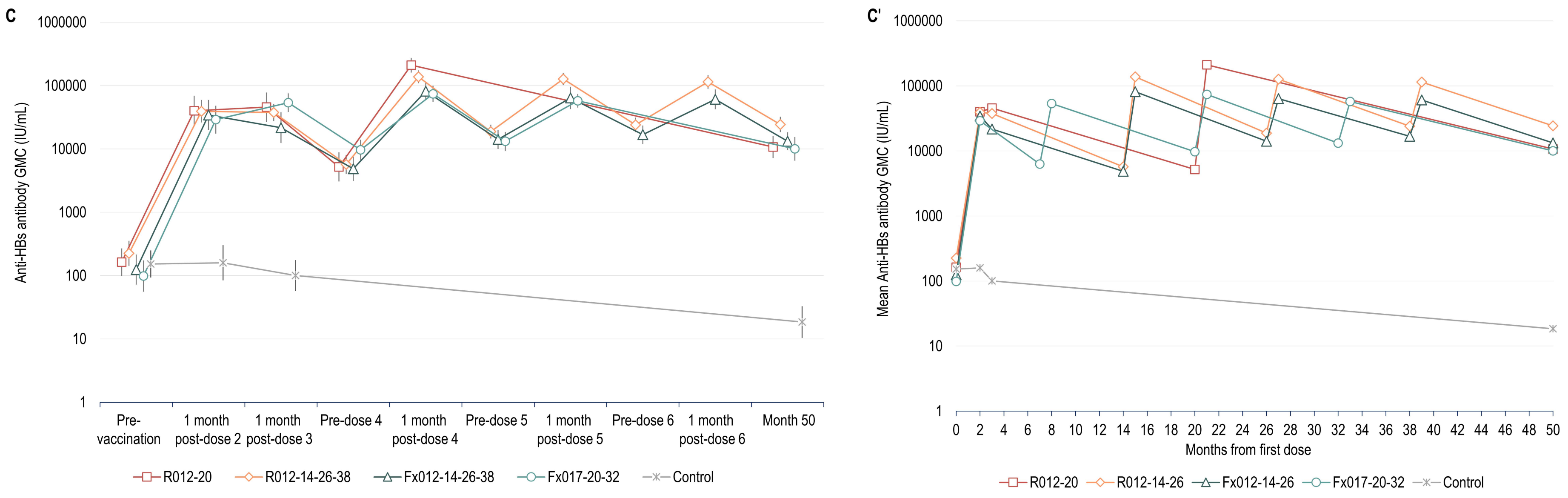

CS, circumsporozoite protein; GMC, geometric mean concentration; EU, enzyme-linked immunosorbent assay units; HBs, anti-hepatitis B surface antigen; IU, international units.

Note: Error bars represent 95% confidence intervals.

Anti-CS antibody concentrations and avidity index (using NH_4SCN as chaotropic agent) were determined by an in-house enzyme-linked immunosorbent assay using plate adsorbed recombinant R32LR antigen, at the Center for Vaccinology (Ghent, Belgium). Anti-HBs antibody concentrations were determined using a chemiluminescence immunoassay (ADVIA Centaur anti–HBs2 /Siemens Healthcare) at GSK Clinical Laboratory Sciences (Rixensart/Wavre, Belgium). GMC calculation was performed by taking the anti-log of the mean of the log transformations (base 10). Antibody concentrations below the cut-off of the assay were given an arbitrary value of half the cut-off for the purpose of GMC calculation (0.5 EU/mL for CS and 6.2 mIU/mL for HBs).

Table S1. Vaccine efficacy against all episodes of clinical malaria over 12 months post-dose 4, dose 5, and dose 6 and from dose 3 up to month 50 (per-protocol set for efficacy)

Time period		Group	Case definition	N	n	T (year)	n/T	VE (95% CI)	p-value
**12 months post-dose 4**									
M14–M26	Overall	Control	Primary	243	334	223·63	1·49	··	··
			Secondary	243	543	215·72	2·52	··	··
		R012-14-26-38	Primary	245	190	218·90	0·87	51% (35; 63)	<0·001
			Secondary	245	329	213·77	1·54	50% (36; 61)	<0·001
		Fx012-14-26-38	Primary	251	198	222·86	0·89	49% (32; 62)	<0·001
			Secondary	251	318	218·31	1·46	50% (36; 61)	<0·001
	Ghana	Control	Primary	136	67	131·18	0·51	··	··
			Secondary	136	97	130·03	0·75	··	··
		R012-14-26-38	Primary	130	25	121·34	0·21	62% (32; 78)	<0·001
			Secondary	130	36	120·95	0·30	62% (39; 77)	<0·001
		Fx012-14-26-38	Primary	129	37	121·73	0·30	44% (5; 67)	0·03
			Secondary	129	54	121·09	0·45	43% (13; 63)	0·01
	Kenya	Control	Primary	107	267	92·44	2·89	··	··
			Secondary	107	446	85·68	5·21	··	··
		R012-14-26-38	Primary	115	165	97·56	1·69	46% (25; 61)	<0·001
			Secondary	115	293	92·82	3·16	44% (25; 58)	<0·001
		Fx012-14-26-38	Primary	122	161	101·13	1·59	51% (31; 65)	<0·001
			Secondary	122	264	97·22	2·72	53% (37; 65)	<0·001
M20–M32	Overall	Control	Primary	231	269	215·48	1·25	··	··
			Secondary	231	429	209·48	2·05	··	··
		R012-20	Primary	230	185	213·88	0·86	43% (21; 59)	<0·001
			Secondary	230	334	208·35	1·60	39% (19; 54)	<0·001
		Fx017-20-32	Primary	261	177	242·53	0·73	48% (30; 61)	<0·001
			Secondary	261	309	237·59	1·30	45% (28; 58)	<0·001
	Ghana	Control	Primary	132	52	133·28	0·39	··	··
			Secondary	132	81	132·18	0·61	··	··
		R012-20	Primary	123	20	124·60	0·16	60% (29; 78)	0·01
			Secondary	123	39	123·91	0·31	53% (23; 72)	0·01
		Fx017-20-32	Primary	137	29	137·36	0·21	47% (11; 68)	0·02
			Secondary	137	57	136·29	0·42	36% (0; 59)	0·05
	Kenya	Control	Primary	99	217	82·20	2·64	··	··
			Secondary	99	348	77·30	4·50	··	··
		R012-20	Primary	107	165	89·28	1·85	34% (1; 55)	0·04
			Secondary	107	295	84·44	3·49	30% (0; 51)	0·05
		Fx017-20-32	Primary	124	148	105·17	1·41	48% (25; 64)	<0·001
			Secondary	124	252	101·30	2·49	50% (29; 64)	<0·001
**12 months post-dose 5**									
M26–M38	Overall	Control	Primary	227	253	207·31	1·22	··	··
			Secondary	227	410	201·43	2·04	··	··
		R012-14-26-38	Primary	232	152	213·17	0·71	57% (39; 69)	<0·001
			Secondary	232	294	207·94	1·41	50% (31; 63)	<0·001
		Fx012-14-26-38	Primary	236	145	216·97	0·67	53% (34; 66)	<0·001
			Secondary	236	254	213·01	1·19	50% (33; 62)	<0·001
	Ghana	Control	Primary	131	52	132·22	0·39	··	··
			Secondary	131	82	131·10	0·63	··	··
		R012-14-26-38	Primary	122	14	119·80	0·12	71% (44; 85)	<0·001

Time period		Group	Case definition	N	n	T (year)	n/T	VE (95% CI)	p-value
			Secondary	122	35	119·01	0·29	62% (30; 79)	0·01
		Fx012-14-26-38	Primary	126	25	122·77	0·20	50% (12; 72)	0·02
			Secondary	126	43	122·14	0·35	47% (12; 68)	0·02
	Kenya	Control	Primary	96	201	75·09	2·68	··	··
			Secondary	96	328	70·33	4·66	··	··
		R012-14-26-38	Primary	110	138	93·37	1·48	49% (23; 66)	0·01
			Secondary	110	259	88·92	2·91	43% (18; 61)	0·01
		Fx012-14-26-38	Primary	110	120	94·20	1·27	54% (31; 69)	<0·001
			Secondary	110	211	90·87	2·32	52% (31; 66)	<0·001
M32–M50*	Overall	Control	Primary	224	323	298·37	1·08	··	··
			Secondary	224	593	288·19	2·06	··	··
		Fx017-20-32	Primary	250	265	335·68	0·79	33% (10; 50)	0·01
			Secondary	250	450	328·72	1·37	38% (19; 52)	<0·001
	Ghana	Control	Primary	128	72	181·00	0·40	··	··
			Secondary	128	106	179·70	0·59	··	··
		Fx017-20-32	Primary	134	46	193·47	0·24	41% (−6; 67)	0·08
			Secondary	134	79	192·24	0·41	31% (–13; 58)	0·14
	Kenya	Control	Primary	96	251	117·37	2·14	··	··
			Secondary	96	487	108·49	4·49	··	··
		Fx017-20-32	Primary	116	219	142·21	1·54	28% (0; 49)	0·05
			Secondary	116	371	136·48	2·72	42% (21; 57)	<0·001
**12 months post-dose 6**									
M38–M50	Overall	Control	Primary	219	191	194·90	0·98	··	··
			Secondary	219	376	187·98	2·00	··	··
		R012-14-26-38	Primary	221	156	202·06	0·77	34% (7; 54)	0·02
			Secondary	221	279	197·45	1·41	40% (20; 55)	<0·001
		Fx012-14-26-38	Primary	225	117	205·22	0·57	50% (28; 65)	<0·001
			Secondary	225	222	201·26	1·10	53% (37; 65)	<0·001
	Ghana	Control	Primary	125	45	117·60	0·38	··	··
			Secondary	125	67	116·76	0·57	··	··
		R012-14-26-38	Primary	118	16	117·02	0·14	66% (26; 84)	0·01
			Secondary	118	33	116·37	0·28	51% (13; 73)	0·02
		Fx012-14-26-38	Primary	122	18	120·19	0·15	63% (22; 82)	0·01
			Secondary	122	30	119·76	0·25	58% (24; 77)	0·01
	Kenya	Control	Primary	94	146	77·31	1·89	··	··
			Secondary	94	309	71·22	4·34	··	··
		R012-14-26-38	Primary	103	140	85·03	1·65	16% (−23; 42)	0·38
			Secondary	103	246	81·08	3·03	34% (8; 52)	0·02
		Fx012-14-26-38	Primary	103	99	85·02	1·16	43% (14; 62)	0·01
			Secondary	103	192	81·50	2·36	51% (30; 65)	<0·001
**Post-dose 3 to M50**									
M2·5–M50	Overall	Control	Primary	265	1010	903·03	1·12	··	··
			Secondary	265	1706	876·54	1·95	··	··
		R012-20	Primary	259	783	848·42	0·92	32% (14; 47)	0·01
			Secondary	259	1449	823·01	1·76	26% (8; 41)	0·01
		R012-14-26-38	Primary	264	632	871·62	0·73	48% (33; 59)	<0·001
			Secondary	264	1125	852·85	1·32	47% (33; 58)	<0·001
		Fx012-14-26-38	Primary	271	619	886·98	0·70	44% (29; 56)	<0·001
			Secondary	271	1055	870·39	1·21	44% (30; 56)	<0·001
	Ghana	Control	Primary	141	216	513·30	0·42	··	··
			Secondary	141	316	509·46	0·62	··	··

Time period		Group	Case definition	N	n	T (year)	n/T	VE (95% CI)	p-value
		R012-20	Primary	134	94	473·19	0·20	58% (35; 73)	<0·001
			Secondary	134	162	470·60	0·34	50% (26; 66)	<0·001
		R012-14-26-38	Primary	135	77	480·40	0·16	66% (47; 78)	<0·001
			Secondary	135	139	478·05	0·29	61% (41; 75)	<0·001
		Fx012-14-26-38	Primary	136	115	487·32	0·24	49% (23; 66)	0·01
			Secondary	136	175	485·05	0·36	46% (22; 63)	0·01
	Kenya	Control	Primary	124	794	389·74	2·04	··	··
			Secondary	124	1390	367·08	3·79	··	··
		R012-20	Primary	125	689	375·23	1·84	11% (–18; 33)	0·41
			Secondary	125	1287	352·41	3·65	6% (−24; 29)	0·68
		R012-14-26-38	Primary	129	555	391·21	1·42	34% (12; 51)	0·01
			Secondary	129	986	374·80	2·63	35% (14; 51)	0·01
		Fx012-14-26-38	Primary	135	504	399·66	1·26	41% (20; 57)	<0·001
			Secondary	135	880	385·33	2·28	43% (25; 57)	<0·001
M7·5–M50	Overall	Control	Primary	237	898	737·86	1·22	··	··
			Secondary	237	1522	714·13	2·13	··	··
		Fx017-20-32	Primary	274	651	846·36	0·77	47% (31; 58)	<0·001
			Secondary	274	1117	828·67	1·35	45% (30; 56)	<0·001
	Ghana	Control	Primary	131	191	436·62	0·44	··	··
			Secondary	131	281	433·17	0·65	··	··
		Fx017-20-32	Primary	141	105	472·23	0·22	54% (29; 70)	<0·001
			Secondary	141	181	469·35	0·39	48% (22; 65)	0·01
	Kenya	Control	Primary	106	707	301·23	2·35	··	··
			Secondary	106	1241	280·96	4·42	··	··
		Fx017-20-32	Primary	133	546	374·13	1·46	42% (21; 57)	<0·001
			Secondary	133	936	359·32	2·60	44% (25; 58)	<0·001

N, number of children in each group contributing to the considered evaluation period; n, number of episodes included in each group; T (year), person-years at risk; n/T, person-year rate in each group; VE, vaccine efficacy; CI, confidence interval; M, month.

Notes: The per-protocol set for efficacy included children who received all three first vaccinations as per protocol and who contributed to efficacy surveillance starting 14 days post-dose 3.

The p-value was calculated using a negative binomial model.

Since the primary objective of the study was not demonstrated, any group comparisons should be interpreted with caution.

*18 months post-dose 5 for group Fx017-20-32.

Table S2. Vaccine efficacy against all episodes of clinical malaria up to month 50 in participants with baseline positive and negative parasitaemia (exposed set)

Group	Case definition	N	n	T (year)	n/T	VE (95% CI)	p-value
**Baseline positive parasitaemia**							
Control	Primary	21	114	72·86	1·56	··	··
R012-20		30	160	98·38	1·63	−1% (−91; 46)	0·97
R012-14-26-38		18	79	60·99	1·30	23% (−63; 64)	0·48
Fx012-14-26-38		14	49	48·42	1·01	12% (−99; 61)	0·75
Fx017-20-32		16	71	48·13	1·48	22% (−56; 61)	0·47
Control	Secondary	21	233	68·34	3·41	··	··
R012-20		30	298	93·08	3·20	2% (−55; 37)	0·95
R012-14-26-38		18	131	59·05	2·22	46% (−4; 72)	0·07
Fx012-14-26-38		14	92	46·77	1·97	20% (−41; 54)	0·44
Fx017-20-32		16	114	46·48	2·45	48% (15; 68)	0·01
**Baseline negative parasitaemia**							
Control	Primary	272	1030	944·94	1·09	··	··
R012-20		268	706	908·15	0·78	41% (23; 54)	<0·001
R012-14-26-38		275	597	944·61	0·63	52% (38; 63)	<0·001
Fx012-14-26-38		290	672	1003·75	0·67	45% (29; 57)	<0·001
Fx017-20-32		295	702	1026·21	0·68	42% (26; 55)	<0·001
Control	Secondary	272	1689	919·86	1·84	··	··
R012-20		268	1288	886·02	1·45	37% (19; 50)	<0·001
R012-14-26-38		275	1070	926·55	1·15	49% (35; 60)	<0·001
Fx012-14-26-38		290	1131	986·23	1·15	44% (29; 56)	<0·001
Fx017-20-32		295	1202	1007·18	1·19	39% (23; 52)	<0·001

N, number of children in each group contributing to the considered evaluation period; n, number of episodes included in each group; T (year), person-years at risk; n/T, person-year rate in each group; VE, vaccine efficacy; CI, confidence interval.

Notes: The p-value was calculated using negative a binomial model.
Since the primary objective of the study was not demonstrated, any group comparisons should be interpreted with caution.

Table S3. Cases averted of clinical malaria (secondary case definition) per 1000 children vaccinated and 1000 full-dose equivalents, by 3-month periods, from month 0 to month 50, overall and by country (exposed set)

	Cumulative number of cases averted (95% CI)							
	Group R012-20		Group R012-14-26-38		Group Fx012-14-26-38		Group Fx017-20-32	
Interval, months	per 1000 children vaccinated	per 1000 full-dose equivalents	per 1000 children vaccinated	per 1000 full-dose equivalents	per 1000 children vaccinated	per 1000 full-dose equivalents	per 1000 children vaccinated	per 1000 full-dose equivalents
Overall								
[0-3]	50·2 (-58·3; 151·8)	17·3 (-20·1; 52·4)	122·9 (23·3; 218·5)	42·0 (8·0; 74·6)	117·8 (20·3; 221·4)	54·7 (9·4; 102·9)	74·5 (-28·6; 170·5)	37·9 (-14·5; 86·8)
[0-6]	194·4 (25·6; 349·1)	67·0 (8·8; 120·4)	309·8 (164·5; 457·1)	105·8 (56·2; 156·1)	322·4 (177·6; 469·3)	149·8 (82·5; 218·0)	201·5 (34·4; 353·1)	101·8 (17·4; 178·4)
[0-9]	313·7 (52·1; 547·1)	108·2 (18·0; 188·7)	487·6 (273·4; 711·1)	166·5 (93·4; 242·8)	473·9 (256·3; 690·5)	220·1 (119·1; 320·8)	345·5 (116·5; 561·9)	160·9 (54·2; 261·7)
[0-12]	407·8 (41·8; 750·3)	140·7 (14·4; 258·8)	748·1 (411·8; 1064·5)	255·5 (140·6; 363·5)	610·1 (304·0; 939·4)	283·4 (141·2; 436·4)	579·5 (249·7; 903·7)	269·9 (116·3; 420·9)
[0-15]	492·8 (-8·7; 970·6)	170·0 (-3·0; 334·8)	1005·7 (565·4; 1428·4)	264·7 (148·8; 376·0)	861·2 (471·2; 1274·9)	370·0 (202·4; 547·7)	832·6 (385·3; 1261·8)	387·7 (179·4; 587·6)
[0-18]	604·7 (-16·9; 1206·8)	208·6 (-5·8; 416·2)	1292·7 (759·6; 1794·4)	340·2 (199·9; 472·3)	1143·2 (648·7; 1666·2)	491·2 (278·7; 715·8)	1138·4 (589·7; 1681·2)	527·0 (273·0; 778·3)
[0-21]	746·6 (46·5; 1412·2)	201·3 (12·5; 380·8)	1459·4 (823·8; 2060·9)	384·1 (216·8; 542·4)	1296·4 (704·5; 1912·8)	557·0 (302·7; 821·8)	1365·2 (734·9; 1997·7)	589·0 (317·1; 861·9)
[0-24]	905·3 (93·2; 1663·4)	243·7 (25·1; 447·8)	1622·8 (865·2; 2353·3)	386·3 (206·0; 560·2)	1434·0 (730·3; 2138·1)	599·0 (305·1; 893·1)	1529·9 (781·1; 2269·8)	659·7 (336·8; 978·8)
[0-27]	957·2 (75·0; 1819·7)	257·4 (20·2; 489·4)	1747·3 (929·2; 2550·0)	377·4 (200·7; 550·8)	1569·3 (792·9; 2340·0)	629·9 (318·3; 939·2)	1658·5 (824·9; 2479·2)	715·2 (355·7; 1069·1)
[0-30]	1049·1 (23·1; 2033·3)	282·2 (6·2; 546·9)	1986·9 (1030·0; 2890·5)	428·3 (222·0; 623·0)	1868·0 (977·2; 2733·2)	749·4 (392·0; 1096·5)	1770·7 (810·1; 2739·5)	738·4 (337·8; 1142·4)
[0-33]	1190·1 (37·6; 2280·5)	320·1 (10·1; 613·3)	2206·9 (1152·4; 3238·5)	475·7 (248·4; 698·0)	2162·4 (1170·9; 3124·6)	867·5 (469·7; 1253·5)	2043·0 (970·7; 3113·4)	823·7 (391·4; 1255·2)
[0-36]	1261·3 (-14·8; 2477·2)	339·2 (-4·0; 666·2)	2355·1 (1184·4; 3533·9)	452·9 (227·7; 679·5)	2362·2 (1273·5; 3416·7)	909·0 (490·0; 1314·8)	2211·2 (1056·1; 3418·0)	889·6 (424·9; 1375·2)
[0-39]	1341·1 (-78·4; 2618·8)	360·7 (-21·1; 704·3)	2552·8 (1269·5; 3807·9)	472·6 (235·0; 705·0)	2602·3 (1418·6; 3738·2)	982·3 (535·5; 1411·0)	2426·0 (1176·7; 3722·7)	976·1 (473·4; 1497·8)
[0-42]	1285·5 (-235·6; 2653·4)	345·8 (-63·4; 713·6)	2665·8 (1272·4; 3986·3)	491·4 (234·5; 734·8)	2766·8 (1523·6; 3950·7)	1042·5 (574·1; 1488·6)	2538·5 (1211·2; 3924·4)	1021·3 (487·3; 1578·9)
[0-45]	1217·4 (-474·5; 2713·6)	327·4 (-127·6; 729·8)	2789·4 (1338·7; 4263·7)	514·2 (246·8; 785·9)	2872·5 (1511·0; 4120·0)	1082·4 (569·3; 1552·4)	2648·6 (1198·3; 4121·1)	1065·6 (482·1; 1658·0)
[0-48]	1308·5 (-392·1; 2876·7)	351·9 (-105·5; 773·7)	2929·4 (1416·9; 4424·1)	540·0 (261·2; 815·5)	3096·3 (1702·7; 4455·1)	1166·7 (641·6; 1678·7)	2809·1 (1308·6; 4339·0)	1130·2 (526·5; 1745·7)
[0-50]	1311·3 (-380·2; 2921·2)	352·7 (-102·3; 785·7)	2950·1 (1440·0; 4448·4)	543·8 (265·4; 820·0)	3054·4 (1651·0; 4394·7)	1150·9 (622·1; 1655·9)	2819·4 (1295·8; 4394·9)	1134·3 (521·3; 1768·2)
Ghana								
[0-3]	7·1 (-56·8; 70·5)	2·4 (-19·5; 24·2)	14·1 (-47·0; 73·9)	4·8 (-16·0; 25·1)	-20·4 (-80·8; 42·9)	-9·3 (-36·8; 19·5)	21·2 (-32·6; 74·2)	10·7 (-16·5; 37·5)
[0-6]	90·4 (-2·9; 179·5)	31·0 (-1·0; 61·6)	105·8 (10·0; 203·4)	35·9 (3·4; 69·0)	43·1 (-53·0; 144·9)	19·6 (-24·1; 65·9)	79·3 (-15·5; 165·6)	40·0 (-7·8; 83·5)
[0-9]	217·3 (90·6; 352·2)	74·5 (31·1; 120·8)	176·8 (38·4; 295·7)	60·0 (13·0; 100·3)	55·8 (-90·9; 209·1)	25·4 (-41·4; 95·1)	110·1 (-28·4; 251·4)	50·8 (-13·1; 115·9)
[0-12]	278·1 (108·2; 446·0)	95·4 (37·1; 153·0)	233·3 (54·3; 401·2)	79·2 (18·4; 136·1)	52·4 (-143·7; 241·6)	23·8 (-65·4; 109·9)	197·1 (27·0; 366·4)	90·9 (12·4; 168·9)
[0-15]	368·7 (178·5; 572·3)	126·5 (61·2; 196·3)	317·4 (112·9; 519·0)	82·5 (29·3; 134·9)	144·5 (-77·6; 358·1)	60·6 (-32·6; 150·3)	304·8 (110·3; 508·4)	140·5 (50·9; 234·3)
[0-18]	536·3 (308·8; 758·0)	184·0 (105·9; 260·0)	516·1 (261·2; 749·3)	134·1 (67·9; 194·7)	277·7 (5·1; 533·9)	116·5 (2·2; 224·1)	461·5 (192·2; 718·1)	212·4 (88·5; 330·6)
[0-21]	601·3 (334·3; 870·3)	160·3 (89·1; 232·0)	612·5 (326·1; 893·2)	159·2 (84·7; 232·1)	285·7 (-39·6; 607·4)	119·9 (-16·6; 254·9)	516·8 (210·1; 831·4)	219·9 (89·4; 353·9)
[0-24]	705·7 (421·6; 991·9)	187·4 (112·0; 263·5)	678·1 (357·0; 979·0)	165·1 (86·9; 238·4)	331·8 (-41·4; 679·4)	136·9 (-17·1; 280·2)	518·3 (164·9; 866·5)	220·3 (70·1; 368·4)
[0-27]	788·6 (470·9; 1119·6)	209·1 (124·9; 296·9)	743·8 (396·4; 1075·4)	159·1 (84·8; 230·0)	363·7 (-51·2; 752·5)	142·1 (-20·0; 294·0)	602·9 (212·6; 982·8)	256·3 (90·4; 417·8)
[0-30]	832·0 (476·4; 1211·1)	220·6 (126·3; 321·1)	841·0 (432·0; 1215·5)	179·1 (92·0; 258·9)	440·9 (4·8; 864·6)	172·1 (1·9; 337·4)	613·7 (177·4; 1031·2)	256·8 (74·3; 431·6)
[0-33]	1033·3 (645·2; 1447·1)	274·0 (171·1; 383·7)	1018·2 (582·4; 1456·8)	216·8 (124·0; 310·3)	608·6 (144·6; 1060·0)	237·5 (56·4; 413·7)	736·7 (221·7; 1233·0)	292·3 (88·0; 489·2)
[0-36]	1052·8 (623·8; 1501·0)	279·2 (165·4; 398·0)	1030·3 (550·4; 1499·1)	201·5 (107·7; 293·2)	633·6 (149·5; 1121·2)	239·9 (56·6; 424·6)	806·6 (268·4; 1327·0)	318·7 (106·0; 524·3)
[0-39]	1109·7 (638·1; 1596·4)	294·2 (169·2; 423·3)	1105·4 (597·7; 1621·8)	202·3 (109·4; 296·8)	712·3 (176·5; 1238·5)	261·1 (64·7; 453·9)	862·9 (272·5; 1422·2)	340·9 (107·6; 561·9)

	Cumulative number of cases averted (95% CI)							
	Group R012-20		Group R012-14-26-38		Group Fx012-14-26-38		Group Fx017-20-32	
Interval, months	per 1000 children vaccinated	per 1000 full-dose equivalents	per 1000 children vaccinated	per 1000 full-dose equivalents	per 1000 children vaccinated	per 1000 full-dose equivalents	per 1000 children vaccinated	per 1000 full-dose equivalents
[0-42]	1156·5 (625·4; 1732·8)	306·7 (165·8; 459·5)	1222·3 (686·4; 1785·9)	221·8 (124·6; 324·1)	833·1 (235·5; 1422·5)	304·3 (86·0; 519·6)	938·3 (311·9; 1549·1)	370·7 (123·2; 612·0)
[0-45]	1114·1 (508·0; 1735·7)	295·4 (134·7; 460·2)	1292·2 (709·1; 1904·2)	234·5 (128·7; 345·6)	845·6 (198·6; 1472·8)	308·9 (72·5; 537·9)	896·7 (216·7; 1565·8)	354·3 (85·6; 618·6)
[0-48]	1176·3 (543·9; 1820·0)	311·9 (144·2; 482·6)	1355·2 (740·9; 2000·9)	246·0 (134·5; 363·1)	970·3 (309·1; 1630·0)	354·4 (112·9; 595·4)	972·2 (280·5; 1673·5)	384·1 (110·8; 661·2)
[0-50]	1178·0 (529·0; 1834·1)	312·4 (140·3; 486·3)	1364·3 (715·4; 2018·7)	247·6 (129·8; 366·4)	911·4 (230·0; 1583·5)	332·9 (84·0; 578·4)	971·2 (264·4; 1664·3)	383·7 (104·5; 657·5)
Kenya								
[0-3]	102·2 (-101·6; 298·4)	35·5 (-35·3; 103·5)	248·7 (73·9; 439·5)	85·5 (25·4; 151·1)	283·4 (114·5; 463·5)	134·3 (54·3; 219·6)	156·4 (-55·4; 362·3)	80·2 (-28·4; 185·8)
[0-6]	319·7 (1·4; 618·8)	110·9 (0·5; 214·7)	542·7 (276·4; 830·3)	186·6 (95·0; 285·4)	647·8 (373·4; 904·9)	307·0 (176·9; 428·8)	369·6 (60·5; 651·8)	187·1 (30·6; 330·0)
[0-9]	436·2 (-52·2; 910·2)	151·3 (-18·1; 315·7)	842·1 (421·0; 1260·1)	289·5 (144·7; 433·2)	965·6 (576·1; 1345·8)	457·6 (273·0; 637·7)	652·9 (229·8; 1069·6)	307·1 (108·1; 503·1)
[0-12]	591·6 (-105·0; 1335·9)	205·2 (-36·4; 463·4)	1359·4 (769·4; 1959·7)	467·3 (264·5; 673·6)	1308·7 (710·0; 1857·3)	620·2 (336·5; 880·1)	1100·1 (487·3; 1678·4)	517·4 (229·2; 789·4)
[0-15]	695·4 (-238·8; 1639·9)	241·2 (-82·8; 568·9)	1841·9 (1070·2; 2632·2)	491·4 (285·5; 702·2)	1786·9 (1042·0; 2575·9)	785·2 (457·9; 1131·9)	1568·0 (768·8; 2355·2)	737·5 (361·6; 1107·7)
[0-18]	765·5 (-334·4; 1886·4)	265·5 (-116·0; 654·4)	2253·1 (1381·3; 3248·0)	601·1 (368·5; 866·5)	2277·5 (1367·8; 3174·6)	1000·8 (601·1; 1395·0)	2088·2 (1118·6; 2993·2)	971·8 (520·6; 1393·0)
[0-21]	1017·7 (-276·2; 2264·9)	277·9 (-75·4; 618·5)	2510·9 (1434·8; 3697·9)	669·9 (382·8; 986·6)	2618·9 (1509·2; 3700·4)	1150·8 (663·2; 1626·1)	2538·7 (1391·6; 3630·2)	1109·8 (608·4; 1587·0)
[0-24]	1258·1 (-211·9; 2721·0)	343·5 (-57·9; 743·0)	2803·5 (1568·6; 4225·2)	651·9 (364·7; 982·4)	2884·0 (1659·4; 4153·3)	1219·3 (701·5; 1755·9)	2922·8 (1611·9; 4187·6)	1277·7 (704·7; 1830·7)
[0-27]	1276·2 (-325·8; 2878·7)	348·5 (-89·0; 786·1)	3002·8 (1655·1; 4645·1)	655·6 (361·3; 1014·1)	3144·7 (1748·2; 4558·4)	1295·7 (720·3; 1878·3)	3115·1 (1665·3; 4498·6)	1361·8 (728·0; 1966·6)
[0-30]	1438·6 (-393·0; 3319·6)	392·8 (-107·3; 906·5)	3431·2 (1823·4; 5299·1)	749·1 (398·1; 1156·9)	3721·5 (2137·8; 5385·4)	1533·4 (880·9; 2219·0)	3372·0 (1671·8; 4916·0)	1401·3 (694·8; 2043·0)
[0-33]	1517·6 (-595·3; 3625·5)	414·4 (-162·6; 990·0)	3717·1 (1931·1; 5795·3)	811·5 (421·6; 1265·2)	4177·1 (2405·8; 6074·6)	1721·1 (991·3; 2503·0)	3835·8 (1914·2; 5629·9)	1570·4 (783·7; 2305·0)
[0-36]	1675·8 (-683·4; 4027·7)	457·6 (-186·6; [illegible]	4051·2 (1999·9; 6289·9)	765·3 (377·8; 1188·2)	4598·3 (2587·7; 6743·0)	1796·9 (1011·2; 2635·0)	4151·0 (2016·3; 6151·9)	1699·5 (825·5; 2518·7)
[0-39]	1802·9 (-724·6; 4325·5)	492·3 (-197·9; [illegible]	4403·8 (2174·6; 6754·9)	825·3 (407·6; 1266·0)	5028·9 (2877·3; 7355·5)	1953·5 (1117·7; 2857·2)	4561·7 (2278·5; 6736·1)	1867·6 (932·8; 2757·9)
[0-42]	1648·3 (-1122·8; 4340·8)	450·1 (-306·6; [illegible]	4521·2 (2160·3; 7005·2)	847·3 (404·9; 1312·9)	5242·6 (2927·6; 7787·7)	2036·5 (1137·2; 3025·1)	4730·0 (2357·7; 7056·6)	1936·6 (965·3; 2889·1)
[0-45]	1564·7 (-1413·3; 4453·8)	427·3 (-385·9; [illegible]	4725·4 (2136·2; 7355·7)	885·6 (400·4; 1378·6)	5463·2 (3000·9; 8194·5)	2122·2 (1165·7; 3183·1)	5028·0 (2452·2; 7461·9)	2058·6 (1004·0; 3055·0)
[0-48]	1674·2 (-1442·8; 4812·9)	457·2 (-394·0; [illegible]	5056·0 (2306·9; 7943·2)	947·6 (432·3; 1488·7)	5968·9 (3365·4; 8878·8)	2318·6 (1307·3; 3448·9)	5320·6 (2564·7; 7987·9)	2178·3 (1050·0; 3270·4)
[0-50]	1713·7 (-1637·0; 5079·4)	468·0 (-447·0; [illegible]	5224·2 (2381·5; 8318·6)	979·1 (446·3; 1559·1)	6137·1 (3432·9; 9182·0)	2383·9 (1333·5; 3566·7)	5488·7 (2697·0; 8260·0)	2247·2 (1104·2; 3381·8)

CI, confidence interval.

Note: The period M0–M32 was first divided in consecutive 3-month intervals. The number of cases averted per 1000 children vaccinated in each of these 3-month intervals was first computed. The last interval stops at the M32 visit date. The earlier intervals are cut by 3-month intervals (30·5 days times 3). To compute the cumulated number of cases averted, the number of cases averted were summed over all the previous 3-month intervals included in the period considered, starting from day 0. The 95% CI are the equal-tail quantiles that correspond to the confidence level (2·5 to 97·5%) of the cumulative number of averted cases computed for each of the 1000 resamples with replacement (bootstrapping). For the calculation of cases averted per 1000 full-dose equivalent doses, the numbers as calculated above were divided to the total number of full-dose equivalents the children in each group could have received at the upper limit of the 3-month interval: for instance, for the total number of cases ([0–32]), the full-dose equivalent doses received at M32 were four in the R012-20 group, five in the R012-14-26 group, 2·6 in the Fx012-14-26 and 2·6 in the Fx017-20-32 group.

Table S4. Distribution of total number of episodes of clinical malaria according to the primary case definition over 38 and 50 months of follow-up, by site and overall (exposed set)

	R012-20			R012-14-26-38			Fx012-14-26-38			Fx017-20-32			Control		
**No·of episodes**	**N**	**n**	**%**	**N**	**n**	**%**	**N**	**n**	**%**	**N**	**n**	**%**	**N**	**n**	**%**
**Primary case definition**															
M0–M38, Overall															
0	298	151	50·7	294	157	53·4	304	141	46·4	311	150	48·2	293	107	36·5
1	298	40	13·4	294	47	16·0	304	63	20·7	311	51	16·4	293	48	16·4
2	298	25	8·4	294	14	4·8	304	23	7·6	311	21	6·8	293	23	7·8
3	298	19	6·4	294	27	9·2	304	15	4·9	311	27	8·7	293	16	5·5
>3	298	63	21·1	294	49	16·7	304	62	20·4	311	62	19·9	293	99	33·8
M0–M38, Ghana															
0	153	115	75·2	151	116	76·8	148	93	62·8	151	105	69·5	147	72	49·0
1	153	22	14·4	151	21	13·9	148	30	20·3	151	24	15·9	147	33	22·4
2	153	8	5·2	151	5	3·3	148	12	8·1	151	8	5·3	147	13	8·8
3	153	4	2·6	151	4	2·6	148	6	4·1	151	8	5·3	147	11	7·5
>3	153	4	2·6	151	5	3·3	148	7	4·7	151	6	4·0	147	18	12·2
M0–M38, Kenya															
0	145	36	24·8	143	41	28·7	156	48	30·8	160	45	28·1	146	35	24·0
1	145	18	12·4	143	26	18·2	156	33	21·2	160	27	16·9	146	15	10·3
2	145	17	11·7	143	9	6·3	156	11	7·1	160	13	8·1	146	10	6·8
3	145	15	10·3	143	23	16·1	156	9	5·8	160	19	11·9	146	5	3·4
>3	145	59	40·7	143	44	30·8	156	55	35·3	160	56	35·0	146	81	55·5
M0–M50, Overall															
0	298	140	47·0	294	145	49·3	304	133	43·8	311	134	43·1	293	94	32·1
1	298	33	11·1	294	46	15·6	304	56	18·4	311	54	17·4	293	55	18·8
2	298	22	7·4	294	17	5·8	304	28	9·2	311	25	8·0	293	21	7·2
3	298	19	6·4	294	20	6·8	304	10	3·3	311	22	7·1	293	15	5·1
>3	298	84	28·2	294	66	22·4	304	77	25·3	311	76	24·4	293	108	36·9
M0–M50, Ghana															
0	153	108	70·6	151	111	73·5	148	90	60·8	151	94	62·3	147	64	43·5
1	153	21	13·7	151	21	13·9	148	28	18·9	151	26	17·2	147	39	26·5
2	153	12	7·8	151	7	4·6	148	13	8·8	151	14	9·3	147	10	6·8
3	153	4	2·6	151	6	4·0	148	5	3·4	151	8	5·3	147	12	8·2
>3	153	8	5·2	151	6	4·0	148	12	8·1	151	9	6·0	147	22	15·0
M0–M50, Kenya															
0	145	32	22·1	143	34	23·8	156	43	27·6	160	40	25·0	146	30	20·5
1	145	12	8·3	143	25	17·5	156	28	17·9	160	28	17·5	146	16	11·0
2	145	10	6·9	143	10	7·0	156	15	9·6	160	11	6·9	146	11	7·5
3	145	15	10·3	143	14	9·8	156	5	3·2	160	14	8·8	146	3	2·1
>3	145	76	52·4	143	60	42·0	156	65	41·7	160	67	41·9	146	86	58·9
**Secondary case definition**															
M0–M38, Overall															
0	298	123	41·3	294	133	45·2	304	110	36·2	311	122	39·2	293	81	27·6
1	298	42	14·1	294	39	13·3	304	55	18·1	311	42	13·5	293	43	14·7
2	298	23	7·7	294	20	6·8	304	34	11·2	311	33	10·6	293	21	7·2

	R012-20			R012-14-26-38			Fx012-14-26-38			Fx017-20-32			Control		
**No·of episodes**	**N**	**n**	**%**	**N**	**n**	**%**	**N**	**n**	**%**	**N**	**n**	**%**	**N**	**n**	**%**
3	298	11	3·7	294	12	4·1	304	22	7·2	311	17	5·5	293	23	7·8
>3	298	99	33·2	294	90	30·6	304	83	27·3	311	97	31·2	293	125	42·7
M0–M38, Ghana															
0	153	100	65·4	151	101	66·9	148	77	52·0	151	95	62·9	147	58	39·5
1	153	24	15·7	151	25	16·6	148	35	23·6	151	19	12·6	147	32	21·8
2	153	14	9·2	151	11	7·3	148	15	10·1	151	18	11·9	147	15	10·2
3	153	5	3·3	151	3	2·0	148	8	5·4	151	7	4·6	147	12	8·2
>3	153	10	6·5	151	11	7·3	148	13	8·8	151	12	7·9	147	30	20·4
M0–M38, Kenya															
0	145	23	15·9	143	32	22·4	156	33	21·2	160	27	16·9	146	23	15·8
1	145	18	12·4	143	14	9·8	156	20	12·8	160	23	14·4	146	11	7·5
2	145	9	6·2	143	9	6·3	156	19	12·2	160	15	9·4	146	6	4·1
3	145	6	4·1	143	9	6·3	156	14	9·0	160	10	6·3	146	11	7·5
>3	145	89	61·4	143	79	55·2	156	70	44·9	160	85	53·1	146	95	65·1
M0–M50, Overall															
0	298	107	35·9	294	127	43·2	304	104	34·2	311	106	34·1	293	72	24·6
1	298	38	12·8	294	33	11·2	304	47	15·5	311	39	12·5	293	47	16·0
2	298	28	9·4	294	16	5·4	304	31	10·2	311	37	11·9	293	17	5·8
3	298	12	4·0	294	16	5·4	304	19	6·3	311	14	4·5	293	17	5·8
>3	298	113	37·9	294	102	34·7	304	103	33·9	311	115	37·0	293	140	47·8
M0–M50, Ghana															
0	153	87	56·9	151	97	64·2	148	73	49·3	151	80	53·0	147	52	35·4
1	153	26	17·0	151	22	14·6	148	32	21·6	151	22	14·6	147	35	23·8
2	153	17	11·1	151	8	5·3	148	16	10·8	151	24	15·9	147	12	8·2
3	153	7	4·6	151	10	6·6	148	8	5·4	151	7	4·6	147	9	6·1
>3	153	16	10·5	151	14	9·3	148	19	12·8	151	18	11·9	147	39	26·5
M0–M50, Kenya															
0	145	20	13·8	143	30	21·0	156	31	19·9	160	26	16·3	146	20	13·7
1	145	12	8·3	143	11	7·7	156	15	9·6	160	17	10·6	146	12	8·2
2	145	11	7·6	143	8	5·6	156	15	9·6	160	13	8·1	146	5	3·4
3	145	5	3·4	143	6	4·2	156	11	7·1	160	7	4·4	146	8	5·5
>3	145	97	66·9	143	88	61·5	156	84	53·8	160	97	60·6	146	101	69·2

N, number of children; n (%), number (percentage) of children with the specified number of episodes; M, month.

Table S5. Unsolicited adverse events occurring within 30 days post-dose 4, 5, and 6 (exposed set)

Group	% (95% CI)			
	**R012-20**	**R012-14-26-38**	**Fx012-14-26-38**	**Fx017-20-32**
Post-dose 4				
N	244	256	266	267
Any unsolicited AE	30·3 (24·6; 36·5)	36·7 (30·8; 42·9)	38·0 (32·1; 44·0)	29·6 (24·2; 35·5)
Grade 3	1·6 (0·4; 4·1)	0·4 (0·0; 2·2)	0·4 (0·0; 2·1)	0·4 (0·0; 2·1)
Related	4·1 (2·0; 7·4)	5·9 (3·3; 9·5)	0·8 (0·1; 2·7)	1·5 (0·4; 3·8)
Grade 3, related	0·8 (0·1; 2·9)	0·0 (0·0; 1·4)	0·0 (0·0; 1·4)	0·4 (0·0; 2·1)
Post-dose 5				
N	··	247	251	259
Any unsolicited AE	··	27·1 (21·7; 33·1)	25·9 (20·6; 31·8)	34·0 (28·2; 40·1)
Grade 3	··	0·4 (0·0; 2·2)	0·8 (0·1; 2·8)	0·4 (0·0; 2·1)
Related	··	4·5 (2·2; 7·8)	0·4 (0·0; 2·2)	1·5 (0·4; 3·9)
Grade 3, related	··	0·0 (0·0; 1·5)	0·0 (0·0; 1·5)	0·0 (0·0; 1·4)
Post-dose 6				
N	··	231	245	··
Any unsolicited AE	··	27·7 (22·0; 34·0)	31·0 (25·3; 37·2)	··
Grade 3	··	0·0 (0·0; 1·6)	0·0 (0·0; 1·5)	··
Related	··	3·0 (1·2; 6·1)	0·4 (0·0; 2·3)	··
Grade 3, related	··	0·0 (0·0; 1·6)	0·0 (0·0; 1·5)	··

AE, adverse event; CI, confidence interval.

Note: All AEs in children not in the reactogenicity subset were reported as unsolicited AEs.
